# Push‐Pull Stiff‐Stilbene: Proton‐Gated Visible‐Light Photoswitching and Acid‐Catalyzed Isomerization

**DOI:** 10.1002/chem.202103052

**Published:** 2021-10-21

**Authors:** David Villarón, Nol Duindam, Sander J. Wezenberg

**Affiliations:** ^1^ Leiden Institute of Chemistry Leiden University Einsteinweg 55 2333 CC Leiden The Netherlands

**Keywords:** molecular switches, photochromism, proton gating, push-pull systems, stiff-stilbene

## Abstract

Donor‐acceptor substituted stiff‐stilbene is shown to undergo isomerization induced by visible light avoiding the need for harmful UV light. This visible‐light photoswitching is inhibited by protonation of the dimethylamino‐donor unit, disrupting the push‐pull character and thus, gating of the photochromic properties is allowed by acid/base addition. Remarkably, the addition of a mild acid also triggers fast thermal back‐isomerization, which is unprecedented for stiff‐stilbene photoswitches usually having a very high energy barrier for this process. These combined features offer unique orthogonal control over switching behavior by light and protonation, which is investigated in detail by ^1^H NMR and UV/Vis spectroscopy. In addition, TD‐DFT calculations are used to gain further insight into the absorption properties. Our results will help elevating the level of control over dynamic behavior in stiff‐stilbene applications.

## Graphical abstract

**Push‐pull stiff‐stilbene**, containing dimethylamino and cyano substituents, undergoes *E‐Z* isomerization upon irradiation with visible light. The photochromic properties can be controlled by acid/base as an orthogonal secondary stimulus. Beside inhibiting the visible‐light triggered isomerization process, acid addition significantly lowers the energy barrier to thermal back‐isomerization.

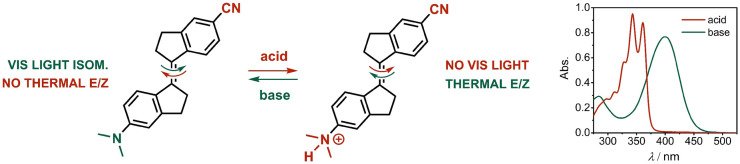

Among molecular photoswitches,^[1]^ stiff‐stilbene is emerging as a powerful tool to construct dynamic functional molecular systems.^[2]^ While originally designed as a prototype for gaining insight into stilbene photoisomerization,^[3]^ its current applications range from the fields of molecular receptors,^[4]^ supramolecular polymers,^[5]^ and catalysis,^[6]^ to lipid bilayer transport.^[7]^ Stiff‐stilbene has two thermally stable states (P‐type photoswitch), high structural rigidity, an efficient photoisomerization process, and undergoes a large geometrical change upon isomerization. However, despite these advantageous properties, they rely on activation by high‐energy UV light causing material degradation and limiting potential application in biology.

The need for activation by UV light is a general drawback of most organic photoswitches and therefore, several strategies have been developed to red‐shift their excitation wavelengths to the visible‐light range.^[8]^ The creation of “push‐pull” systems by attachment of electron‐donating and ‐withdrawing groups has, for instance, proven successful for azobenzene switches^[9]^ and overcrowded alkene‐based molecular motors.^[10]^ In addition to visible‐light‐induced switching, there is rapidly growing interest in the development of dual‐stimuli responsive photochromic systems. For example, by gating the photochromic properties using redox processes,^[11]^ ion binding,^[12]^ acid/base,^[13]^ or macrocyclic hosts that bind/thread the molecular photoswitch.^[14]^ Furthermore, acid addition was found to elevate the thermal isomerization barrier of certain azobenzene derivatives^[15]^ and conversely, was used to catalyze thermal ring‐opening of diarylethene in order to release the energy stored in the photogenerated form.^[16]^ Such dual‐responsiveness offers an improved level of control over isomerization behavior and therefore, will likely lead to the development of more complex and sophisticated dynamic functions, as well as the improvement of molecular opto‐electronic (memory) devices. Yet, orthogonality between the different stimuli, which is an essential prerequisite, remains highly challenging to achieve.^[17]^

Toward visible‐light switching of stiff‐stilbene, we decided to investigate the influence of donor‐acceptor substituents on the photochromic properties. Therefore, compound (*E*)‐**1** containing a dimethylamino and cyano group in *para*‐position to the double bond was synthesized.^[18]^ Herein, we demonstrate that isomerization of **1** can be triggered by visible light (405/455 nm) and that reversible protonation of the dimethylamino‐donor unit using mild acid/base allows gating of this process (Scheme 1). Moreover, the acid was found to catalyze thermally‐activated *Z*→*E* back‐isomerization and hence, by incorporating donor‐acceptor substituents, unprecedented control over stiff‐stilbene switching behavior is achieved.

**Scheme 1:**
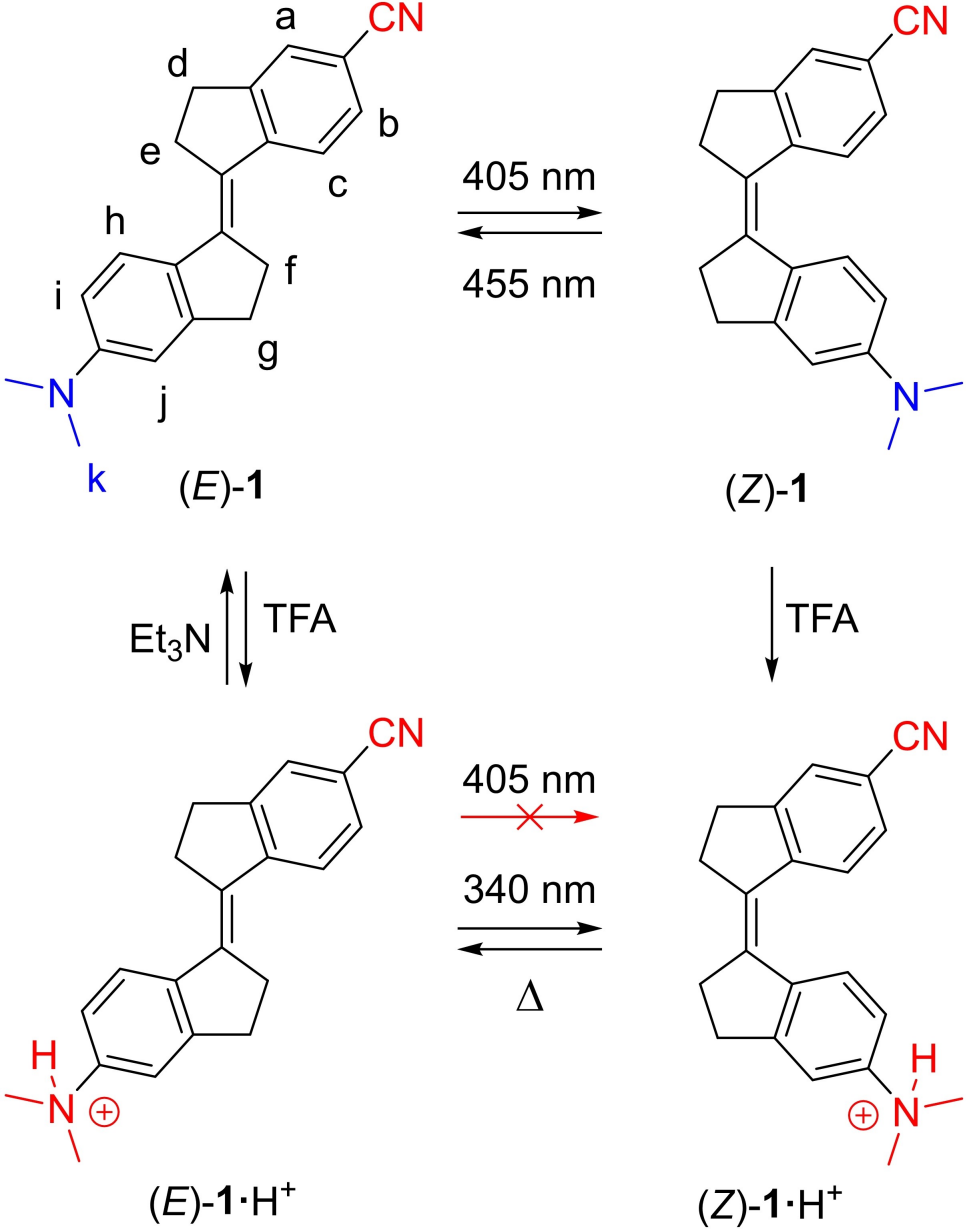
Light‐induced and acid/base‐controlled isomerization steps of donor‐acceptor substituted stiff‐stilbene **1**.

Desymmetrization of stiff‐stilbene has previously been achieved through mixed McMurry[^4d^, ^4f^] and Barton‐Kellogg^[19]^ reactions. In 1992, Lapouyade and co‐workers first described compound (*E*)‐**1**, which was obtained via a mixed coupling of 5‐bromo‐1‐indanone and 5‐dimethylamino‐1‐indanone followed by cyanation using copper cyanide.^[18]^ Unfortunately, when we attempted a mixed McMurry coupling with these indanones, only a trace amount of the desired heterocoupled product was observed in the ^1^H NMR spectrum of the crude reaction mixture, which mainly consisted of 5,5′‐dibromo‐subsituted stiff‐stilbene (Figure S13 in the Supporting Information). Therefore, we devised an alternative strategy for stiff‐stilbene desymmetrization based on mono‐functionalization of the 5,5′‐dibromo product (see Scheme 2 and the Supporting Information for synthetic details and characterization). First, McMurry homocoupling of 5‐bromo‐1‐indanone afforded a mixture of (*E*)‐**2** and (*Z*)‐**2**. The (*E*)‐isomer could be isolated by precipitation and was submitted to Buchwald‐Hartwig amination to obtain mono‐amine (*E*)‐**3**. Subsequent methylation using methyl iodide under basic conditions, followed by Pd‐catalyzed cyanation using zinc cyanide, gave the desired push‐pull stiff‐stilbene (*E*)‐**1**.

**Scheme 2:**
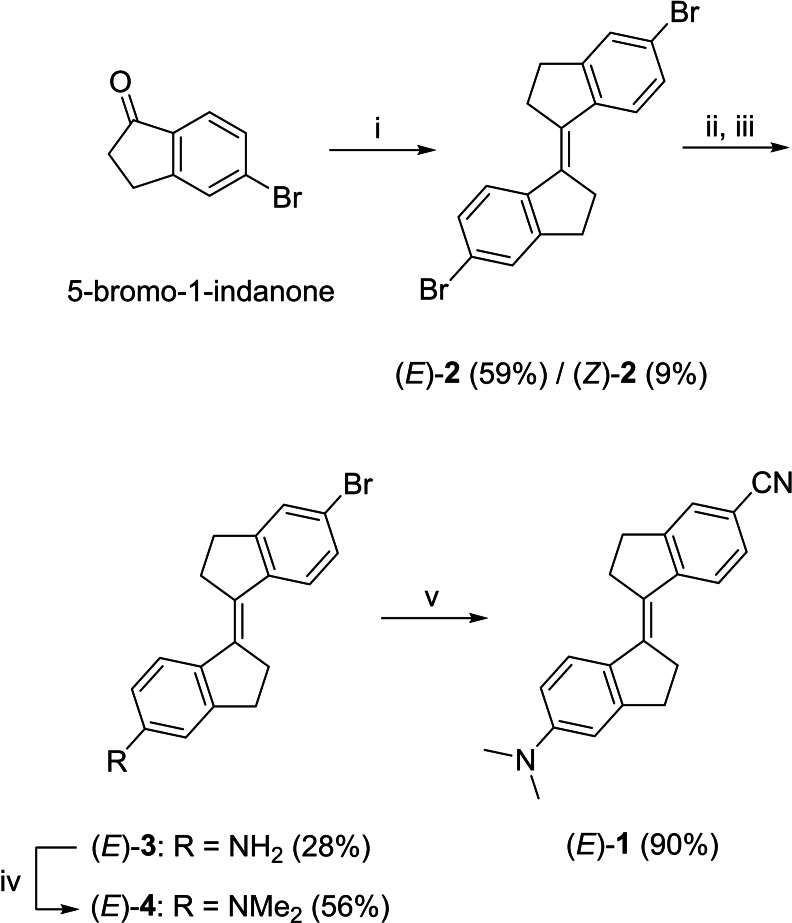
Synthesis of push‐pull stiff‐stilbene (*E*)‐**1**: (i) Zn, TiCl_4_, THF, reflux; (ii) benzophenone imine, Pd(OAc)_2_, DPPF, NaOtBu, toluene, 90 °C; (iii) 2 M aqueous HCl, THF; (iv) MeI, K_2_CO_3_, DMF; (v) Zn(CN)_2_, tBuXPhos, tBuXPhos‐Pd‐G3, DMF/H_2_O (99 : 1 v/v). Note that only the (*E*)‐isomer of **2** is drawn in the scheme for convenience.

The photoisomerization properties of (*E*)‐**1** were initially examined by UV/Vis spectroscopy in MeCN solution. The UV/Vis absorption spectrum (Figure 1 and Figure S14 in the Supporting Information) shows a maximum at *λ*=398 nm and is similar to the one reported by Lapouyade et al.^[18]^ The overall absorption is considerably red‐shifted with respect to unsubstituted stiff‐stilbene (*λ*_max_=342 nm, 324 nm, see Figure S23 in the Supporting Information)^[3]^ revealing a smaller HOMO‐LUMO gap. Irradiation with 405 nm light resulted in a decrease of the absorption maximum and a slight bathochromic shift, which can be ascribed to *E*→*Z* isomerization (Figure 1A).[^2^, ^3^, ^4^, ^5^, ^6^, ^7^] Consecutive irradiation with shorter wavelengths (i. e., 385 nm and 365 nm) led to a slightly larger decrease in absorption, implying higher conversion toward the (*Z*)‐isomer. The reverse UV/Vis spectral changes were observed when the sample was subsequently irradiated with 455 nm light, demonstrating that the (*E*)‐isomer can be regenerated, albeit not quantitatively. In all cases, irradiation was continued until the absorption spectrum did not change further meaning that the photostationary states (PSS) had been reached. Importantly, the clear isosbestic point at *λ*=425 nm confirms unimolecular conversion (Figure S15 in the Supporting Information) and no signs of photodegradation were noted when 405/455 nm irradiation was repeated multiple times (Figure 1B). The quantum yield for 405 nm triggered *E*→*Z* isomerization (Φ_*E*→*Z*_) was determined as 27.2±0.26 % by using potassium ferrioxalate as actinometer (see Figures S16–17 and the Supporting Information for details) With the molar absorptivities and PSS ratio at 405 nm (see below), the quantum yield for the reverse process (Φ_*Z*→*E*_) was calculated as 29.9±0.29 %.

**Figure 1:**
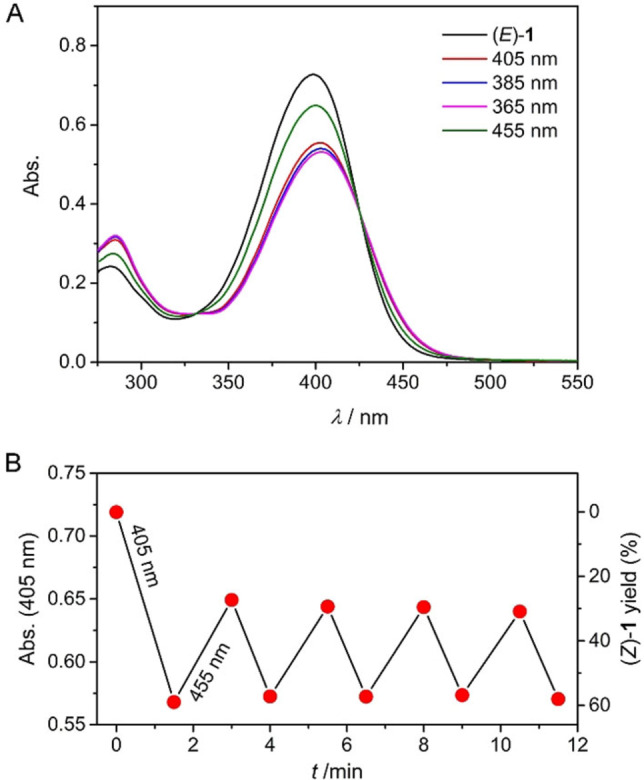
(A) UV/Vis spectral changes of (*E*)‐**1** (2.5×10^−5^ M in MeCN) upon sequential 405 nm, 385 nm, 365 nm, and 455 nm irradiation. (B) Plot of the change in absorption at *λ*=405 nm and (*Z*)‐**1** amount upon multiple 405/455 nm switching cycles starting with (*E*)‐**1** (2.5×10^−4^ M in MeCN).

Next, the *E*→*Z* isomerization process was monitored with ^1^H NMR spectroscopy. Irradiation with either 405 nm or 365 nm light of a solution of (*E*)‐**1** in MeCN‐*d_3_* led to the appearance of a new set of ^1^H NMR signals, which were assigned to (*Z*)‐**1** (Figure 2 and Figure S18 in the Supporting Information). By relative integration, the PSS_405_ and PSS_365_ ratios were determined as 41 : 59 (*E/Z*) and 34 : 66 (*E/Z*), respectively, in line with the slightly larger UV/Vis absorption decrease when 365 nm light was used instead of 405 nm light. Using these PSS ratios and the change in absorbance at *λ*=400 nm, the PSS_455_ was estimated as 74 : 26 (*E/Z*) and the PSS_385_ ratio as 37 : 63 (*E/Z*) (Table S1 in the Supporting Information).

**Figure 2:**
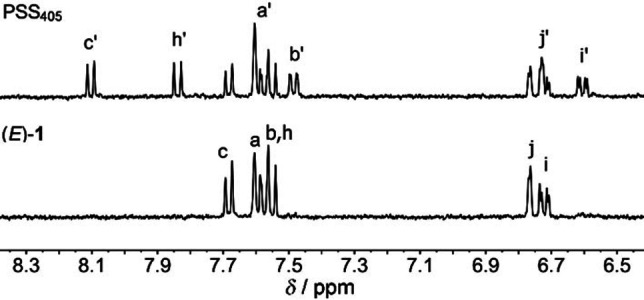
Aromatic region in the ^1^H NMR spectrum of (*E*)‐**1** (2.8 mM in MeCN‐*d*_3_, bottom) and the photostationary state mixture obtained upon 405 nm irradiation (top). The proton assignment is based on COSY and NOESY spectra, see Scheme 1 for the atom labeling.

The absorption properties of (*E*)‐**1** were further investigated using TD‐DFT calculations [B3LYP/6‐31G(d,p), IEFPCM MeCN], which are in reasonable agreement with experimental data,^[20]^ i. e. *λ*_max_ (exp)=398 nm and *λ*_max_ (calcd)=428 nm (see Figure S27 and the Supporting Information for details). The optical gap is significantly red‐shifted with respect to unsubstituted stiff‐stilbene (Figures S26–31 in the Supporting Information) and the S_0_→S_1_ excitation was identified as a pure HOMO‐LUMO π‐π* transition with a clear intramolecular charge‐transfer component. That is, electron density migrates from the dimethylamino‐donor to the cyano‐acceptor upon excitation to the LUMO (Figure 3A). Furthermore, the LUMO displays antibonding character with regard to the central alkene illustrating that the double bond character is decreased in the excited state.

**Figure 3:**
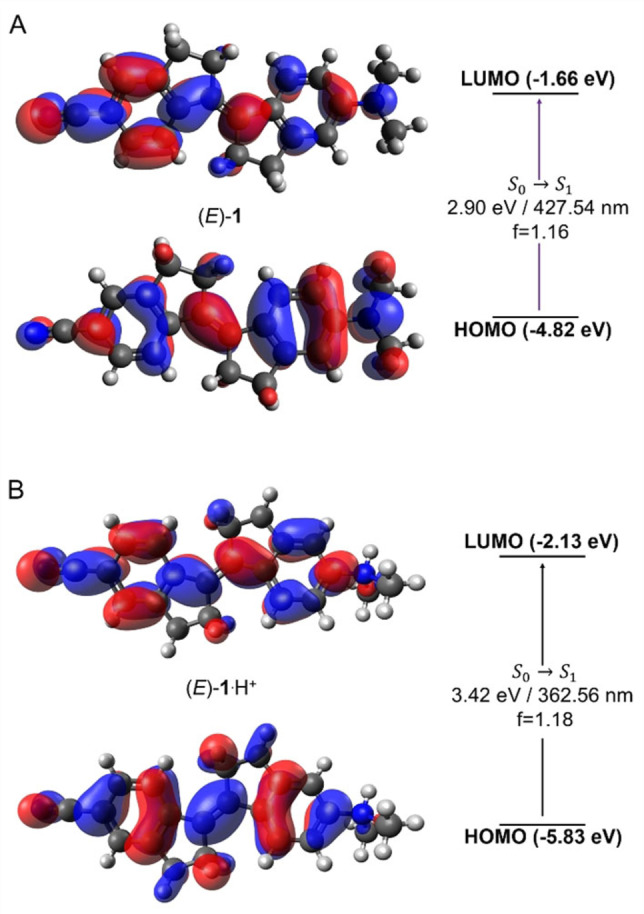
Visualization of the relevant frontier molecular orbitals alongside the predicted and dominating (>99 %) S_0_→S_1_ HOMO‐LUMO transitions (f=oscillator strength) for (A) (*E*)‐**1** and (B) (*E*)‐**1**⋅H^+^. Please note that the S_0_→S_1_ excitation energies (optical gaps) are shown on top of the arrows, between the HOMO and LUMO levels.

We then became interested to investigate the influence of dimethylamine protonation as it would disrupt the donor‐acceptor character and hence, alter absorption ‐ and usable excitation ‐ wavelength. That would potentially allow proton‐gated visible‐light photoswitching. Indeed, TD‐DFT calculations predicted a sharp blue‐shift of 0.52 eV in the optical gap (i. e. S_0_→S_1_ excitation energy) of (*E*)‐**1** upon protonation of the dimethylamino‐donor unit, and inspection of the S_0_→S_1_ transition revealed the loss of intramolecular charge transfer (see Figure 3B and Figure S29 in the Supporting Information).

In line with these predictions, addition of TFA to a solution of (*E*)‐**1** in MeCN led to a large hypsochromic shift, showing absorption maxima at much shorter wavelengths (*λ*_max_=361 nm and 343 nm, Figure 4A and Figure S20 in the Supporting Information), with the shape of the absorption spectrum quite similar to unsubstituted stiff‐stilbene (see Figure S23 in the Supporting Information). As the protonated species (*E*)‐**1⋅**H^+^ did not absorb light beyond *λ*=400 nm, irradiation with 405 nm light did not cause photoisomerization as evident from the absence of spectral changes. Nevertheless, irradiation of the protonated species with 340 nm light led to a decrease in absorption and a small bathochromic shift, indicating formation of the (*Z*)‐**1⋅**H^+^ isomer (Figure 4A and Figure S21 in the Supporting Information).

**Figure 4:**
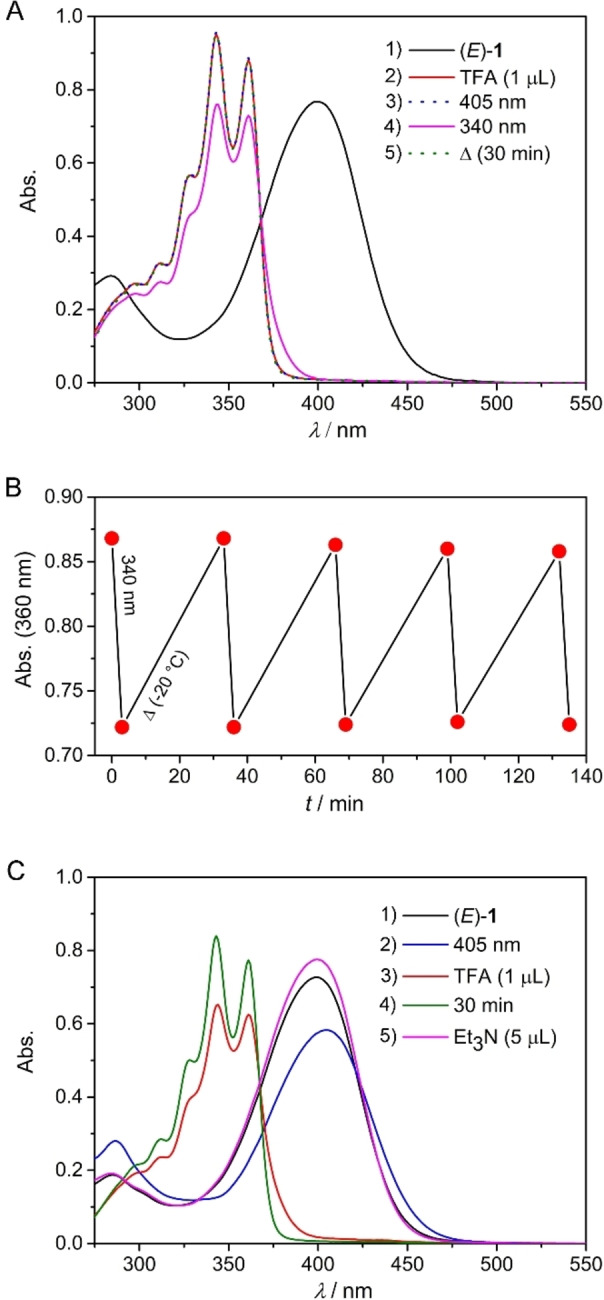
(A) UV/Vis spectral changes starting with (1) (*E*)‐**1** (2.5×10^−5^ M in MeCN) recorded at −20 °C upon successive (2) addition of TFA (1 μL, 0.05 % v/v), (3) 405 and (4) 340 nm irradiation, and (5) equilibration for 30 min. (B) Plot of the change in absorption at *λ*=360 nm upon sequential photo‐ and thermal isomerization steps. (C) Spectral changes starting with (1) (*E*)‐**1** upon successive (2) 405 nm irradiation, (3) TFA (1 μL, 0.05 % v/v) addition, (4) equilibration for 30 min, and (5) Et_3_N (5 μL, 0.25 % v/v) addition.^[24]^

Whereas stiff‐stilbene is known to have a very high energy barrier to thermal isomerization (Δ^≠^ *G*°=180 kJ mol^−1^ and *t*_1/2_=10^9^ years at 300 K),^[21]^ to our surprise, the photogenerated (*Z*)‐**1⋅**H^+^ species spontaneously converted back to (*E*)‐**1⋅**H^+^ as apparent from the recovery of the initial absorption band over time (Figure 4A and Figure S21 in the Supporting Information). A similar observation was made by ^1^H NMR spectroscopy where addition of TFA to a PSS_365_ *E/Z* mixture led to rapid formation of (*E*)‐**1⋅**H^+^ (see Figure S25 in the Supporting Information). These photo‐ and thermal isomerization steps could be repeated several times without signs of fatigue (Figure 4B). Importantly, under similar conditions, unsubstituted stiff‐stilbene did not show such acid‐catalyzed isomerization behavior (Figure S23 in the Supporting Information) and moreover, only a minor change in PSS_405_ (*E*)‐**1**/(*Z*)‐**1** ratio was noted in the absence of TFA for a sample that stood in the dark for 24 h (Figure S19 in the Supporting Information).

Acid‐catalyzed *Z*→*E* isomerization of stilbene has been known since long time, but requires very strong concentrated acid (50–60 % H_2_SO_4_).^[22]^ The proposed mechanism involves double bond protonation to generate a carbenium ion intermediate, which is the rate‐limiting step. Very recently, norbornene‐fused stilbene was reported to isomerize under much milder conditions, i. e., in the presence of TFA the reaction was completed within 2 h at rt in CH_2_Cl_2_ whereas it took several days in MeCN.^[23]^ Also here a carbenium ion intermediate was suggested. In the present case, by determining the rate constant for conversion of (*Z*)‐**1⋅**H^+^ into (*E*)‐**1⋅**H^+^ at −20 °C using the absorption decay at 375 nm (see Figure S22 in the Supporting Information), we calculated a half‐life (*t*_1/2_) at this temperature of 20.7 s, corresponding to a Gibbs free energy barrier (Δ^≠^ *G*°) of 68.8 kJ mol^−1^. TFA‐mediated *Z*→*E* isomerization proceeds thus very rapidly at this low temperature. Protonation of the double bond is tentatively predicted to play an important role here as well, however, the precise mechanism and the effect of other electron‐donating and ‐withdrawing groups still need further investigation.

The serendipitous discovery of this acid‐catalyzed *Z*→*E* isomerization allows orthogonal switching by light and protonation as illustrated in Scheme 1. While the conversion of (*Z*)‐**1** back into (*E*)‐**1** is not quantitative when 455 nm irradiation is used (PSS_455_=74 : 26 *E/Z*), the (*E*)‐isomer can be fully regenerated by sequential protonation and deprotonation as demonstrated in Figure 4C. In short, by starting with a solution of (*E*)‐**1**, initial 405 nm irradiation led to an absorption decrease as a result of the formation of (*Z*)‐**1**. When then TFA was added to the PSS_405_ *E/Z* mixture, the whole absorption spectrum became blue‐shifted because of dimethylamine protonation and resulting loss of donor‐acceptor character. The absorption increase that followed by letting the solution stand for 30 min revealed thermal conversion of (*Z*)‐**1⋅**H^+^ into (*E*)‐**1⋅**H^+^. Final deprotonation using Et_3_N gave back the UV/Vis absorption spectrum of the initial (*E*)‐isomer (which slightly changed in the presence of this base)^[24]^ and thus, completed the isomerization cycle. Importantly, the addition of acid can efficiently release the (visible light) energy stored in the photogenerated (*Z*)‐form. In addition and as shown above, the (*Z*)‐**1⋅**H^+^ species is alternatively accessible through TFA addition to (*E*)‐**1** followed by 340 nm irradiation while 405 nm irradiation did not have any effect here. Visible‐light‐triggered isomerization can thus be switched on/off by using Et_3_N and TFA, i. e. the photochromic properties are gated by acid/base.

In summary, we have synthesized a donor‐acceptor substituted stiff‐stilbene via a newly developed desymmetrization method based on mono‐functionalization. This stiff‐stilbene can be isomerized by visible light (405/455 nm) avoiding the need for harmful UV light, which is normally used to induce this process. The donor‐acceptor character – and with that the visible light absorption – is lost by protonation of the dimethylamino‐donor by using acid and is restored by treatment with base, offering proton‐gated visible‐light excitation. Remarkably, unprecedented TFA‐catalyzed thermal *Z*→*E* isomerization was observed, while the energy barrier to this process is known to be very high for unsubstituted stiff‐stilbene.^[21]^ This acid‐catalyzed process would permit the use as a T‐type photoswitch in “out‐of‐equilibrium” systems^[25]^ and moreover, the orthogonal light and acid/base responsiveness will offer higher levels of control in materials and biological applications.^[2]^

## Conflict of interest

The authors declare no conflict of interest.

## Supporting information

As a service to our authors and readers, this journal provides supporting information supplied by the authors. Such materials are peer reviewed and may be re‐organized for online delivery, but are not copy‐edited or typeset. Technical support issues arising from supporting information (other than missing files) should be addressed to the authors.

Supporting Information
