# Supplementary material for: Push‐Pull Stiff‐Stilbene: Proton‐Gated Visible‐Light Photoswitching and Acid‐Catalyzed Isomerization
Source: Chemistry. 2021 Oct 21;27(69):17346–50. doi: 10.1002/chem.202103052 (PMC9298359; doi:10.1002/chem.202103052)
Supplement: Supplementary file 1 — Supporting Information [file CHEM-27-17346-s001.pdf]

Chemistry–A European Journal

Supporting Information

Push-Pull Stiff-Stilbene: Proton-Gated Visible-Light Photoswitching and Acid-Catalyzed Isomerization

David Villarón, Nol Duindam, and Sander J. Wezenberg*

Table of Contents:

Experimental section S2

^{1}H and ^{13}C NMR Spectra of new compounds S7

^{1}H NMR analysis of mixed McMurry reaction S19

UV-Vis photoisomerization studies S20

Quantum yield determination S21

^{1}H NMR photoisomerization studies S23

UV-Vis studies in the presence of acid S25

^{1}H NMR studies in the presence of acid S28

Geometry optimizations by DFT S29

Time-dependent DFT calculations S36

References S39

Experimental section

General methods and materials:

THF, MeCN, and toluene were dried using a Pure Solve 400 solvent purification system from Innovative Technology. Dry DMF was purchased from Acros Organics and DMSO-*d*6 , MeCN-*d*3 and $CDCl_3$ were purchased from Eurisotop. DMSO-*d*6 was stored under N_2 over molecular sieves (4Å) and $CDCl_3$ was filtered over basic Al_2O_3 to remove DCl. The degassing of the solvents was carried out by purging with N_2 for 15 min, unless noted otherwise. (*E*)-2,2',3,3'-tetrahydro-1,1'-biindenylidene [(*E*)-**6**] was prepared according to a procedure reported in the literature.[1] All other chemicals were commercial products and were used without further purification. Column chromatography was performed using silica gel (SiO_2) purchased from Screening Devices BV (Pore diameter 55-70 Å, surface area 500 m^2g^{-1}) and neutral aluminum oxide (Al_2O_3) purchased from Fluka Analytical. Thin-layer chromatography (TLC) was carried out on aluminum sheets coated with silica 60 F254 and neutral aluminum oxide obtained from Merck. Compounds were visualized with UV light (254 nm) or by staining with potassium permanganate. ^{1}H and ^{13}C NMR spectra were recorded on Bruker AV 400 and Bruker 500 Ultra Shield instruments at 298 K unless indicated otherwise. Chemical shifts (δ) are denoted in parts per million (ppm) relative to residual protiated solvent (DMSO-*d*6: for ^{1}H detection, δ = 2.50 ppm; for ^{13}C detection, δ = 39.52 ppm; $CDCl_3$: for ^{1}H detection, δ= 7.26 ppm; for ^{13}C detection, δ = 77.16 ppm; MeCN-*d*3: for ^{1}H detection, δ= 1.94 ppm; for ^{13}C detection, δ = 1.32 and 118.26 ppm). The splitting pattern of peaks is designated as follows: s (singlet), d (doublet), t (triplet), q (quartet), p (quintet), h (septet), m (multiplet), br (broad). High-resolution mass spectrometry (ESI-MS) was performed on a Thermo Scientific Q Exactive HF spectrometer with ESI ionization. IR spectra were recorded on a Perkin Elmer Spectrum Two FT-IR spectrometer. The wavenumber (ν) is in units of reciprocal centimeters (cm^{-1}) and the intensity (ν = cm^{-1}) is designated as follows: s (strong), m (medium), w (weak), very w (very weak), br (broad), and sh (shoulder). Melting points were determined, with a Büchi M560 apparatus. UV-Vis spectra were recorded on an Agilent Cary 8454 spectrometer using 1 cm or 1 mm quartz cuvettes. Irradiation of UV-Vis and NMR samples was carried out using Thorlab model M340F3 (0.85 mW), M365F1 (3.0 mW), M385F1 (9.0 mW), M405F1 (3.0 mW), M455F3 LEDs (17 mW) positioned at a distance of 1 cm to the sample. Longpass filters with cut-on wavelengths (λ_{cut-on}) of 280±5 nm and 360±5 nm were acquired from Newport Corporation.

(*E*)/(*Z*)-5,5'-Dibromo-2,2'tetrahydro-1,1'-biindenylidene [(*E*)/(*Z*)-2]:[2,3]

First, $TiCl_4$ (4.18 mL, 37.9 mmol) was slowly added to a vigorously stirred suspension of Zn powder (4.95 g, 75.7 mmol) in THF (38 mL) under N_2 atmosphere. The resulting mixture was stirred at reflux for 2 h and cooled to rt. Subsequently, 5-bromo-1-indanone (4.01 g, 19.0 mmol) was added to the black suspension and the mixture was stirred at reflux for another 16 h, cooled to rt and treated with a saturated aqueous NH_4Cl solution (50 mL) and extracted with $CHCl_3$ (4 × 80 mL). The volume of the combined organic layers was reduced to 30 mL and the resulting precipitate was filtered off and air-dried to obtain (*E*)-**2** (2.21 g, 59%) as a yellow solid. R_f = 0.48 (SiO_2, pentane); m.p. 273 – 275 °C ([3]: 269 – 271 °C); ^{1}H NMR (400 MHz, $CDCl_3$, (*E*)-isomer assignment based on the NOESY spectrum in Figure S4): δ = 7.46–7.42 (m, 2H), 7.41 (s, 2H), 7.36 (dd, *J* = 8.0, 1.8 Hz, 2H), 3.11 (s, 8H) ppm; too insoluble for a ^{13}C NMR measurement; IR (ATR): ν = 2945 (w), 2922 (m), 2877 (w), 2832 (very w), 1852 (w), 1743 (w), 1581 (m), 1560 (w, sh), 1465 (m), 1450 (m), 1422 (m), 1407 (br. m), 1297 (m), 1279 (m), 1249 (m), 1203 (m), 1170 (m), 1115 (w), 1069 (m), 868 (s), 818 (m), 782 (s) cm^{-1} (consistent with reported data[3]). The filtrate was concentrated and purification by column chromatography (SiO_2, pentane), afforded (*Z*)-**2** (0.35 g, 9%) as a yellow solid. R_f = 0.40 (SiO_2, pentane); m.p. 183 – 185 °C; ^{1}H NMR (400 MHz, $CDCl_3$): δ = 7.82 (d, *J* = 8.0 Hz, 2H), 7.42 (d, *J* = 1.6 Hz, 2H), 7.25 (dd, *J* = 8.0, 1.6 Hz, 2H), 3.02–2.73 (m, 8H) ppm; ^{13}C NMR (400 MHz, $CDCl_3$): δ = 150.7, 139.4, 134.7, 128.8, 128.5, 124.3, 120.9, 34.8, 30.5 ppm; IR (ATR): ν = 2937 (m), 2901 (very w), 2835 (w), 1716 (br. w), 1638 (m), 1583 (m), 1564 (m), 1459 (s), 1441 (s), 1410 (s), 1325 (w), 1302 (w), 1259 (m), 1194 (m), 1169 (m), 1099 (w), 1060 (m), 999 (w), 953 (w), 864 (s), 829 (s), 809 (s) 793 (s), 713 (m) cm^{-1}.

(*E*)-5'-Bromo-2,2',3,3'-tetrahydro-[1,1'-biindenylidene]-5-amine[(*E*)-3]:

Compound (*E*)-**2** (0.46 g, 1.2 mmol), palladium(II) acetate (15 mg, 0.067 mmol), DPPF (56 mg, 0.10 mmol) and sodium *tert*-butoxide (0.11g, 1.2 mmol) were placed in a schlenk tube and brought under a N_2 atmosphere by three vacuum/N_2 cycles. Then, degassed toluene (6 mL) and benzophenone imine (0.20 mL, 1.2 mmol) were added and the mixture was stirred at 90 °C for 20 h, after which it was diluted with water (5 mL) and extracted with $CHCl_3$ (3 × 5 mL). The combined organic layers were dried over Na_2SO_4 and concentrated. Purification by column chromatography (SiO_2, 0.3% Et_3N in pentane) afforded the mono-imine intermediate as an orange solid. This intermediate was redissolved in THF (16 mL) and subsequently a 2M aqueous HCl solution (8 mL) was added. The mixture was stirred at rt for 1.5 h, treated with a saturated

aqueous $NaHCO_3$ solution (pH ~10) and extracted with EtOAc (3 × 10 mL). The combined organic layers were dried over Na_2SO_4 and concentrated. The product was precipitated in Et_2O, filtered off and air-dried to afford (*E*)-**3** (106 mg, 28%) as a brown solid. Some yield of the imine intermediate was lost in the column purification process, possibly due to hydrolysis. R_f = 0.71 (SiO_2, CH_2Cl_2); m.p. 203 – 205 °C; ^1H NMR (400 MHz, $CDCl_3$): δ = 7.35–7.05 (m, 4H), 6.51 (s, 1H), 6.45 (d, J = 8.0 Hz, 1H), 3.61 (br. s, 2H), 3.13–2.70 (m, 8H) ppm; ^{13}C NMR (400 MHz, $CDCl_3$): δ = 149.4, 148.9, 146.2, 142.8, 136.4 134.0, 130.5, 129.4, 128.1, 125.6, 125.2, 119.8, 113.8, 111.4, 32.1, 31.8, 31.1, 31.0 ppm; IR (ATR): ν = 3423 (w), 3306 (w), 3199 (br. w), 3016 (very w), 2936 (w), 2851 (w), 1624 (m, sh), 1594 (s), 1574 (s), 1487 (s), 1466 (m), 1440 (m), 1409 (m), 1337 (m), 1320 (m), 1268 (br. m), 1252 (m, sh), 1206 (very w), 1178 (w), 1116 (w), 1069 (m), 1043 (m), 960 (very w), 933 (w), 863 (m), 849 (m), 825 (s), 792 (s), 659 (br. m), 565 (m) cm^{-1}; HRMS (ESI) *m/z:* 326.0533 ($[M]^+$, calcd for $C_{18}H_{16}BrN^+$: 326.0539).

(*E*)-5'-Bromo-N,N-dimethyl-2,2',3,3'-tetrahydro-[1,1'-biindenylidene]-5-amine [(*E*)-4]: Compound (*E*)-**3** (50 mg, 0.15 mmol) and K_2CO_3 (0.13 g, 0.92 mmol) were dissolved in dry DMF (0.6 mL). Then, MeI (19 µL, 0.31 mmol) was added and the mixture was stirred at 50 °C for 15 h. Subsequently, the mixture was treated with water (5 mL) and extracted with $CHCl_3$ (3 × 5 mL). The combined organic layers were dried over Na_2SO_4, concentrated and purification by column chromatography (SiO_2, pentane/CH_2Cl_2 7:3) afforded (*E*)-**4** (30 mg, 56%) as a brown solid. R_f = 0.50 (SiO_2, Pentane/CH_2Cl_2 8:2); m.p. 178 – 180 °C; ^1H NMR (400 MHz, $CDCl_3$): δ = 7.48 (d, J = 8.0 Hz, 1H), 7.42–7.36 (m, 2H), 7.32 (dd, J = 8.0, 0.8 Hz, 2H), 6.69 (d, J = 2.5 Hz, 1H), 6.66 (dd, J = 8.0, 2.4 Hz, 1H), 3.15–3.04 (br. m, 8H), 3.00 (s, 6H) ppm; ^{13}C NMR (400 MHz, $CDCl_3$): 150.3, 149.2, 148.8, 143.0, 136.7, 131.9, 129.9, 129.4, 128.0, 125.4, 125.0, 119.5, 111.2, 108.6, 40.8, 32.1, 31.7, 31.4, 31.0 ppm; IR (ATR): ν = 3663 (very w), 3418 (w), 2923 (br. m), 2852 (w, sh), 2807 (w, sh), 1732 (w), 1713 (w), 1598 (s), 1570 (m), 1498 (m), 1463 (w, sh), 1450 (m), 1432 (w, sh), 1406 (w), 1354 (s), 1328 (m, sh), 1299 (m), 1286 (w), 1275 (w), 1252 (w), 1230 (m), 1196 (m), 1173 (m), 1105 (m), 1066 (s), 1044 (s), 1006 (m), 967 (m), 864 (m), 844 (m), 818 (s), 788 (s), 748 (m), 726 (s), 658 (w), 567 (m) cm^{-1}; HRMS (ESI) *m/z:* 354.06774 ($[M]^+$, calcd for $C_{20}H_{20}BrN^+$: 354.0852).

(*E*)-5'-(Dimethylamino)-2,2',3,3'-tetrahydro-[1,1'-biindenylidene]-5-carbonitrile [(*E*)-1]: In a schlenk tube, compound (*E*)-**4** (13 mg, 37 µmol), *t*BuXPhos Pd G3 (9.5 mg, 12 µmol), *t*BuXPhos (8.5 mg, 20 µmol) and $Zn(CN)_2$ (8.7 mg, 74 µmol) were brought under Argon by three vacuum/Argon cycles. Degassed DMF/H_2O (0.6 mL, 99:1 v/v), obtained by purging with Argon for 30 min, was added and the resulting solution was heated to 70 °C for 15 h. Then, EtOAc (5 mL) was added and the organic phase was washed with brine, dried over Na_2SO_4 and concentrated. Purification by column chromatography (SiO_2, pentane/CH_2Cl_2 1:1) afforded (*E*)-**1** (10 mg, 90%) as a yellow solid. R_f = 0.40 (SiO_2, pentane/CH_2Cl_2 1:1); m.p. 229 – 231 °C; ^{1}H NMR (400 MHz, CDCl3): δ = 7.57 (d, *J* = 8.0 Hz, 1H), 7.53-7.46 (m, 3H), 6.69 (d, *J* = 2.5 Hz, 1H), 6.66 (dd, *J* = 8.0, 2.5 Hz, 1H), 3.17-3.09 (br. m, 8H), 3.02 (s, 6H) ppm; ^{13}C NMR (400 MHz, $CDCl_3$): δ = 150.7, 149.9, 148.7, 147.1, 140.6, 131.2, 130.7, 129.3, 128.1, 125.9, 123.9, 120.2, 111.2, 108.3, 40.7, 32.4, 31.6, 31.4, 30.8 ppm; IR (ATR): ν = 2923 (br. m), 2849 (2, sh), 2218 (s), 1684 (w), 1621 (very w, sh), 1607 (m), 1575 (s), 1504 (s), 1477 (m), 1437 (m), 1421 (w, sh), 1362 (s), 1333 (m), 1293 (m), 1279 (w), 1262 (m), 1226 (m), 1202 (s), 1113 (s), 1064 (w), 1045 (s), 967 (w, sh), 957 (m), 919 (very w, sh), 907 (s), 879 (w), 838 (m), 820 (s), 785 (s), 729 (m), 718 (m), 592 (m) cm^{-1}; HRMS (ESI) *m/z:* 300.1622 ([M]$^+$, calcd for $C_{21}H_{20}N_2^+$: 300.1621).

5-(Dimethylamino)-2,3-dihydro-1*H*-inden-1-one (5):
5-Amino-1-indanone (0.40 g, 2.7 mmol) and K_2CO_3 (2.25 g, 16.3 mmol) were brought under a N_2 atmosphere in a Schlenk tube by three vacuum/N_2 cycles. Then, DMF (9 mL) and MeI (0.67 mL, 11 mmol) were added and the resulting mixture was stirred at 55 °C for 16 h. The mixture was treated with water (10 mL) and extracted with $CHCl_3$ (3 × 25 mL). The combined organic layers were dried over Na_2SO_4 and concentrated. Purification by column chromatography (neutral Al_2O_3, CH_2Cl_2/ EtOAc 1:1) afforded **5** (0.24 g, 50%) as a pale yellow solid. R_f = 0.46 (SiO_2, CH_2Cl_2/EtOAc 1:1); m.p. 124 – 126 °C; ^{1}H NMR (400 MHz, $CDCl_3$): δ = 7.63 (d, *J* = 8.6 Hz, 1H), 6.67 (dd, *J* = 8.4, 2.4 Hz, 1H), 6.57 (d, *J* = 2.4 Hz, 1H), 3.08 (s, 6H), 3.05–3.00 (m, 2H), 2.66–2.60 (m, 4 H) ppm; ^{13}C NMR (400 MHz, $CDCl_3$): δ = 205.0, 158.2, 155.1, 125.8, 125.3, 111.8, 106.9, 40.5, 36.6, 26.0 ppm; IR (ATR): ν.= 2921 (m), 2816 (w, sh), 1672 (s), 1591 (br. s), 1518 (m, sh), 1436 (s), 1361 (s), 1348 (s), 1292 (s), 1252 (very w, sh), 1231 (m), 1192 (m), 1151 (w, sh), 1097 (s), 1064 (s), 1029 (m), 984 (w), 869 (w), 835 (m), 809 (s) cm^{-1}; HRMS (ESI) *m/z:* 176.1068 ([M+H]$^+$, calcd for $C_{11}H_{14}NO^+$: 176.1070).

Attempted mixed McMurry reaction of 5-bromo-1-indanone and compound 5:

First, $TiCl_4$ (0.18 mL, 1.7 mmol) was slowly added to a vigorously stirred suspension of Zn powder (0.22 g, 3.4 mmol) in THF (1 mL) under a N_2 atmosphere. The resulting mixture was stirred at reflux for 2 h and cooled to rt. Then, 5-bromo-1-indanone (94 mg, 0.45 mmol) and compound **5** (66 mg, 0.38 mmol) were added to the black suspension and the mixture was stirred at reflux for another 16 h, cooled to rt and treated with a saturated aqueous NH_4Cl (10 mL) solution and extracted with $CHCl_3$ (3 × 20 mL). The combined organic layers were dried over Na_2SO_4 and concentrated, to afford a crude residue (see Figure S13 for ^{1}H NMR analysis).

^{1}H and ^{13}C NMR Spectra of new compounds

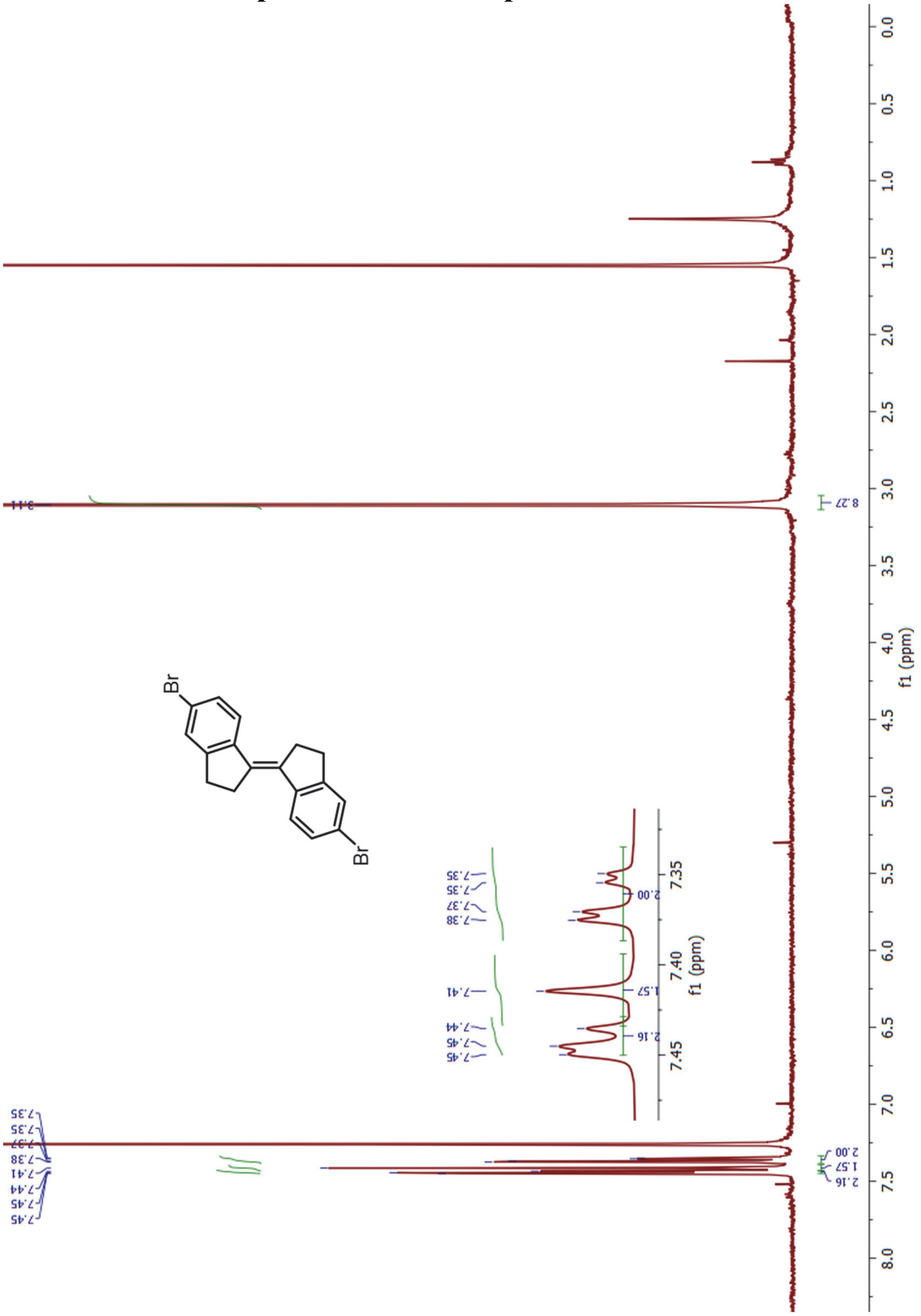

Figure S1. 400 MHz ^{1}H NMR spectrum of (*E*)-**2** measured at 298 K in $CDCl_3$.

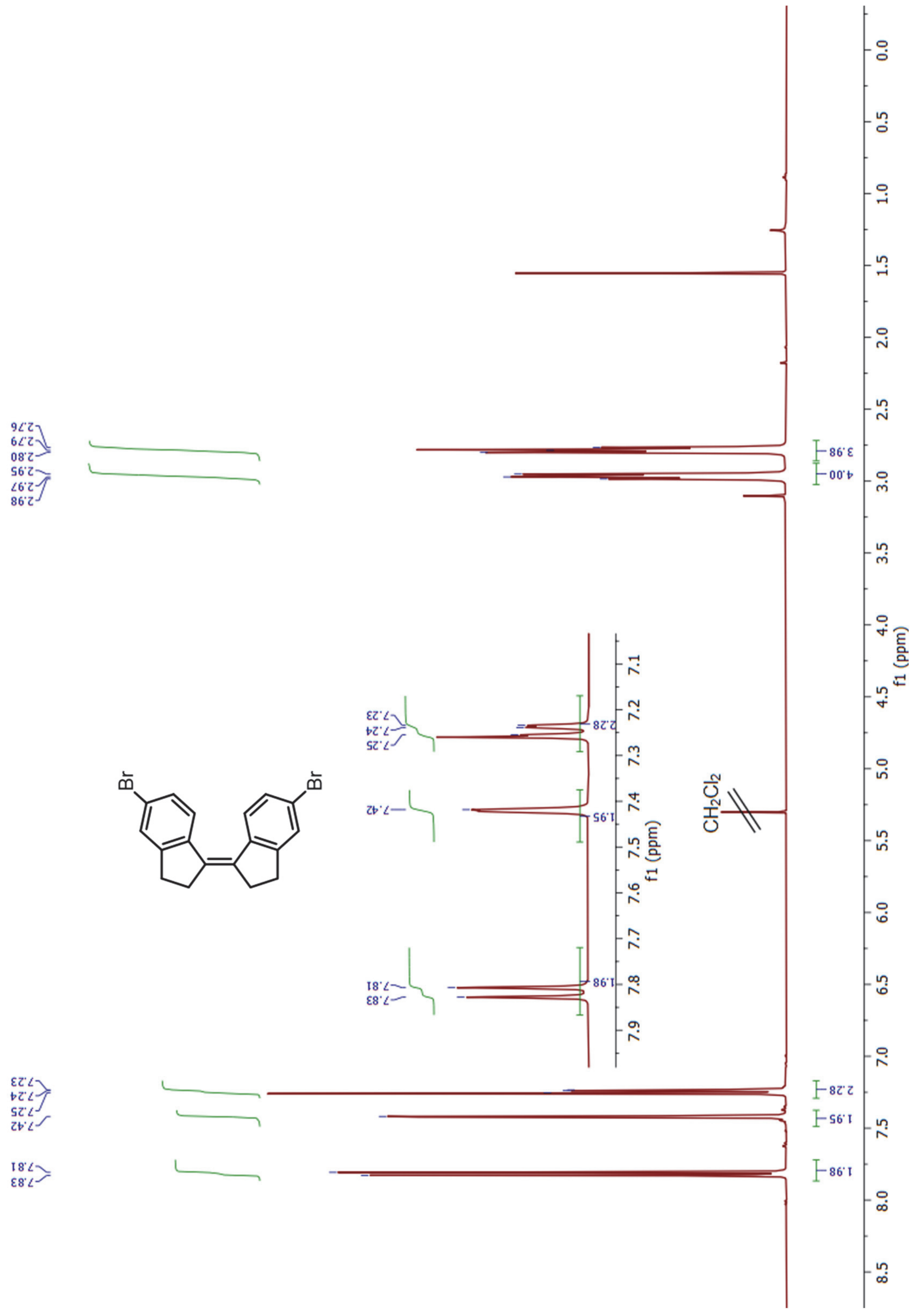

Figure S2. 400 MHz ^{1}H NMR spectrum of (*Z*)-**2** measured at 298 K in $CDCl_3$.

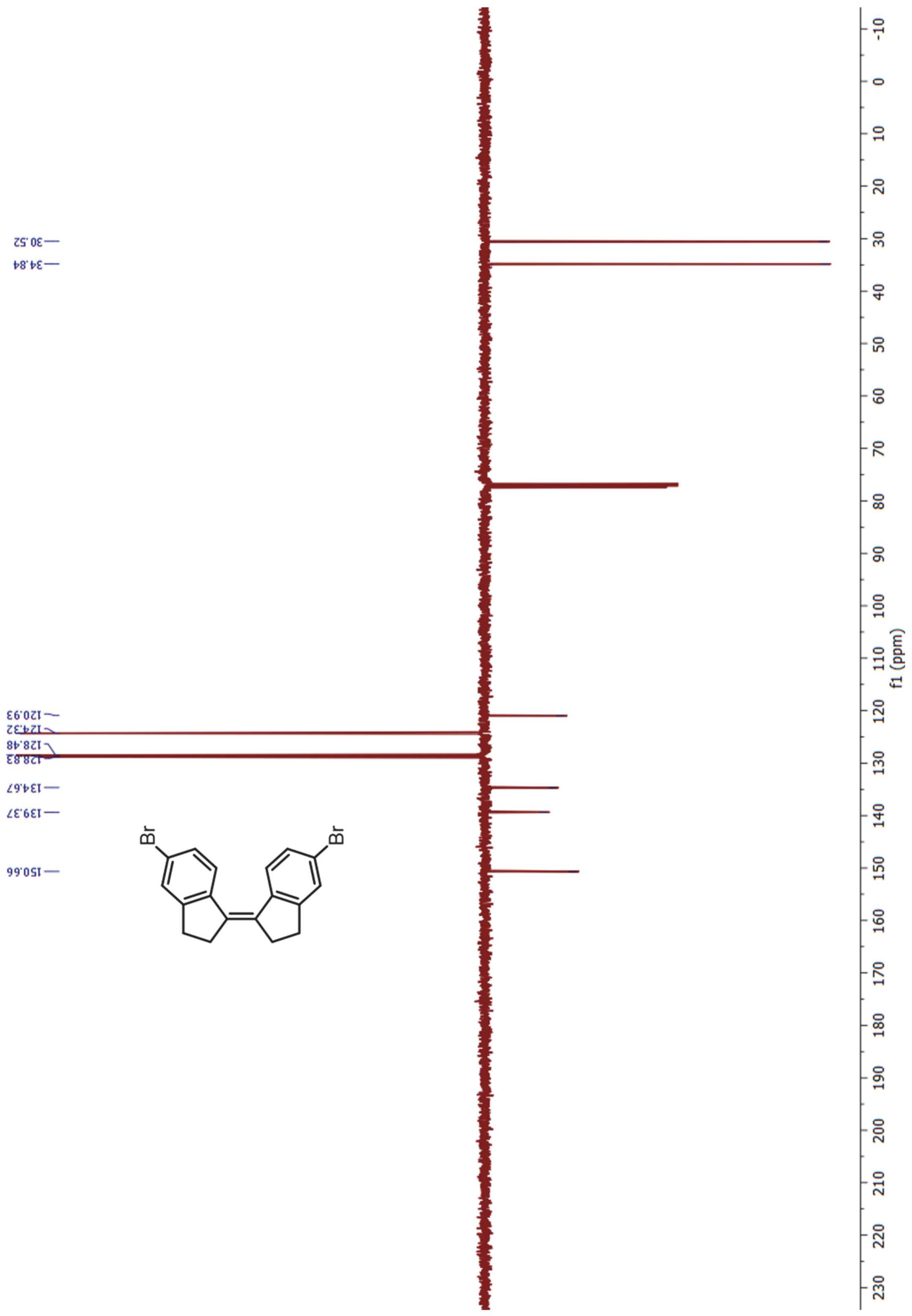

Figure S3. 400 MHz ^{13}C NMR APT spectrum of (*Z*)-**2** measured at 298 K in $CDCl_3$: CH and CH_3 signals positive and quaternary carbon and CH_2 signals negative.

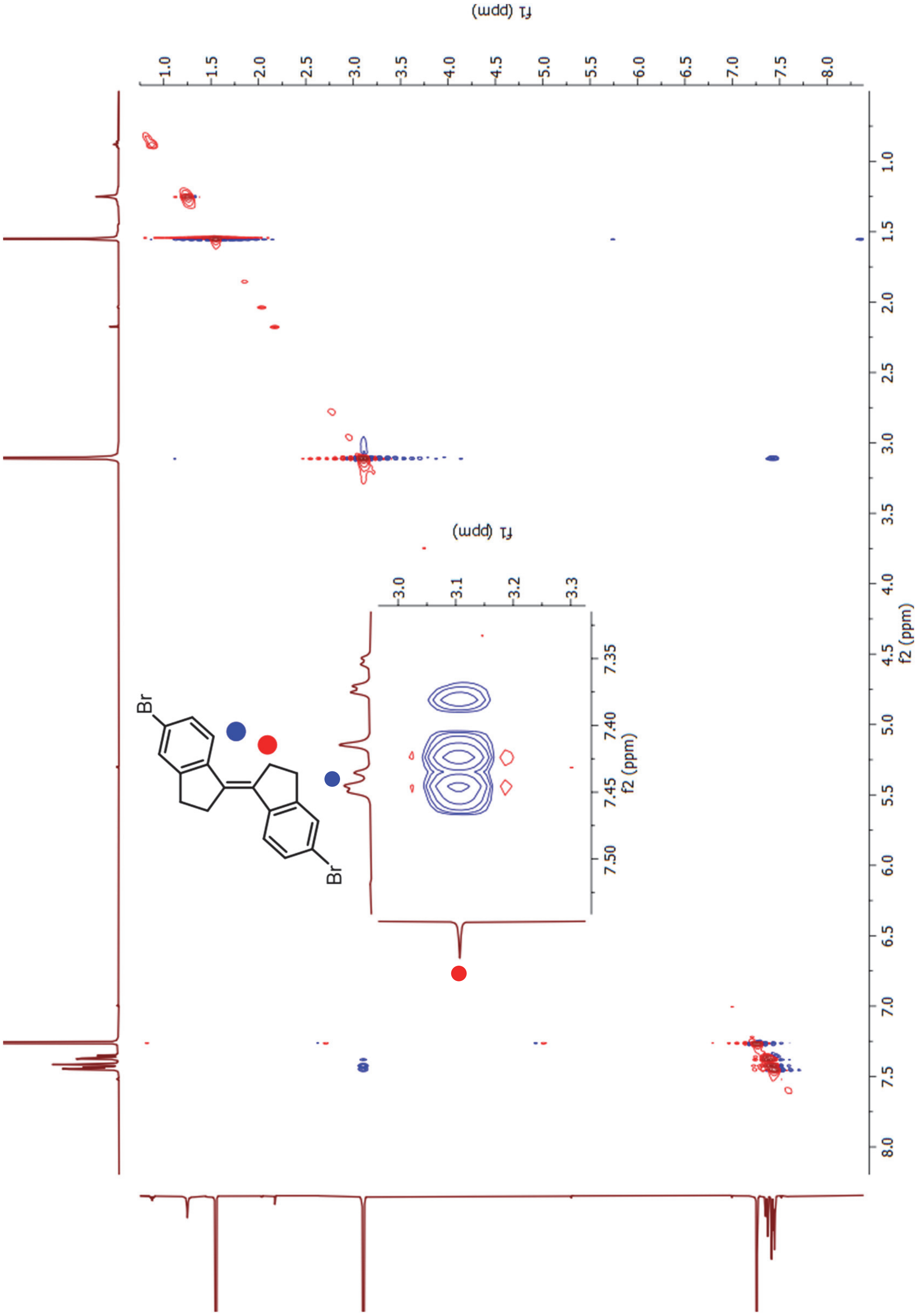

Figure S4. NOESY spectrum of (*E*)-**2** at 298 K in $CDCl_3$.

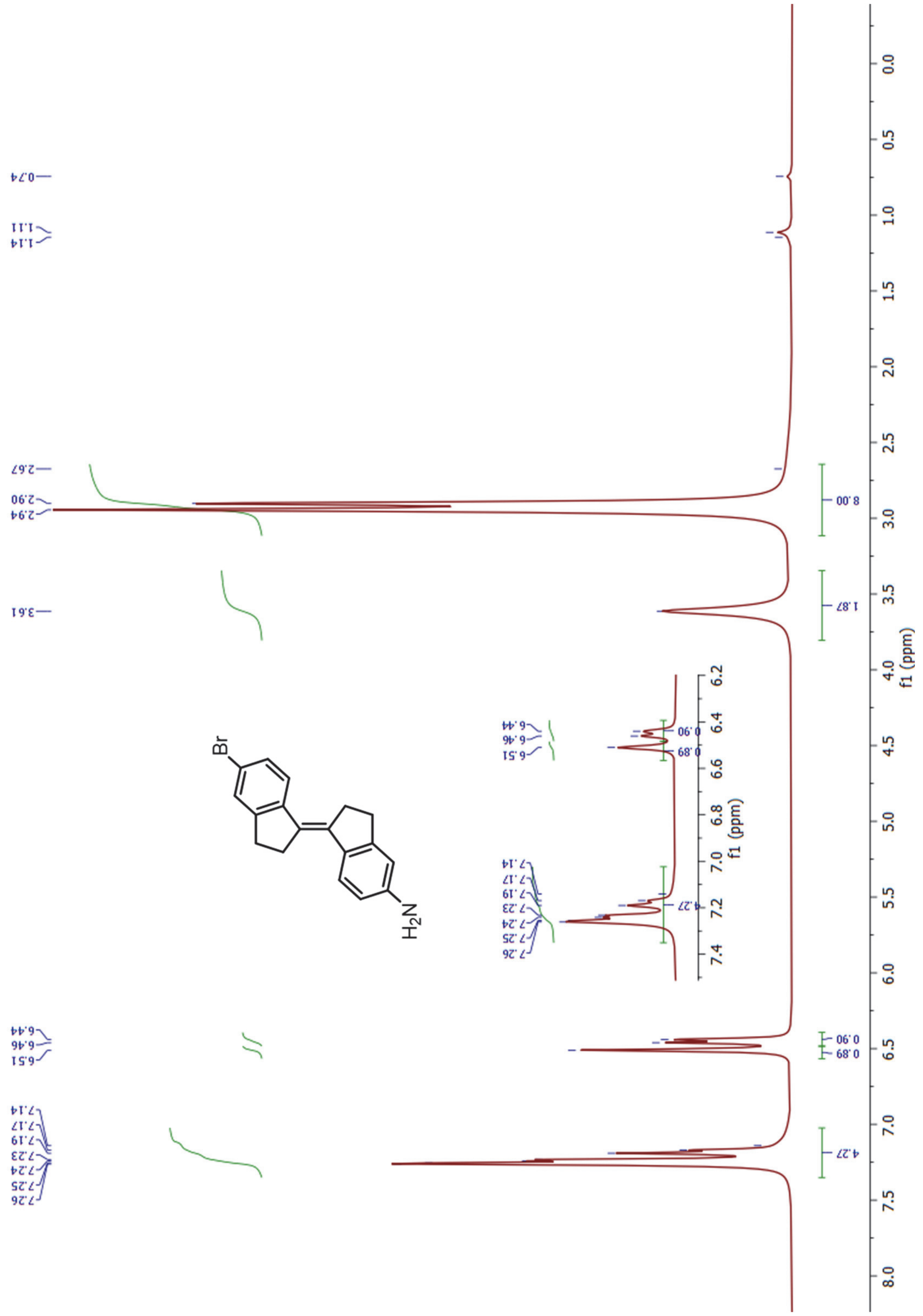

Figure S5. 400 MHz ^{1}H NMR spectrum of (*E*)-**3** measured at 298 K in $CDCl_3$.

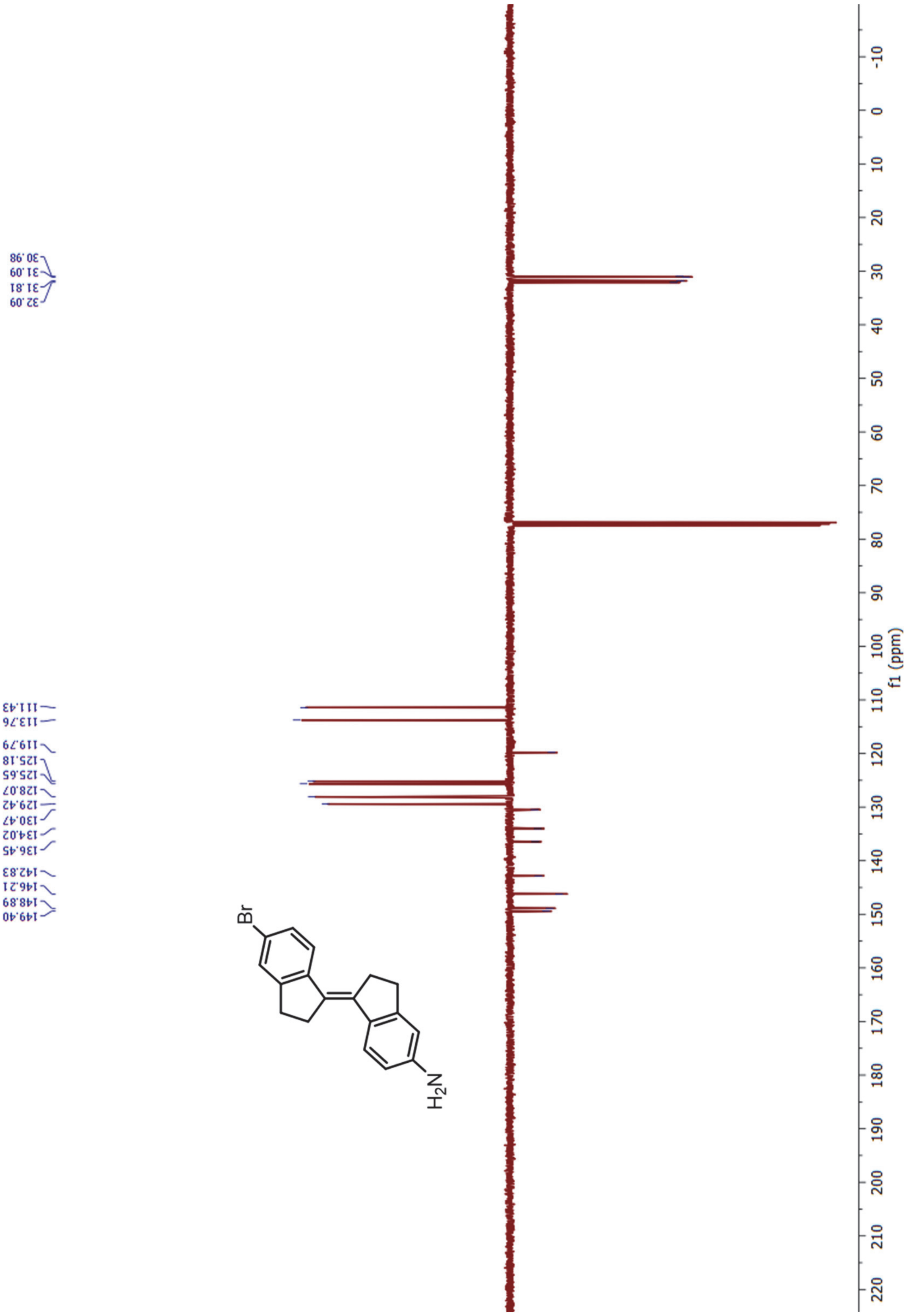

Figure S6. 400 MHz ^{13}C NMR APT spectrum of (*E*)-**3** measured at 298 K in $CDCl_3$: CH and CH_3 signals positive and quaternary carbon and CH_2 signals negative.

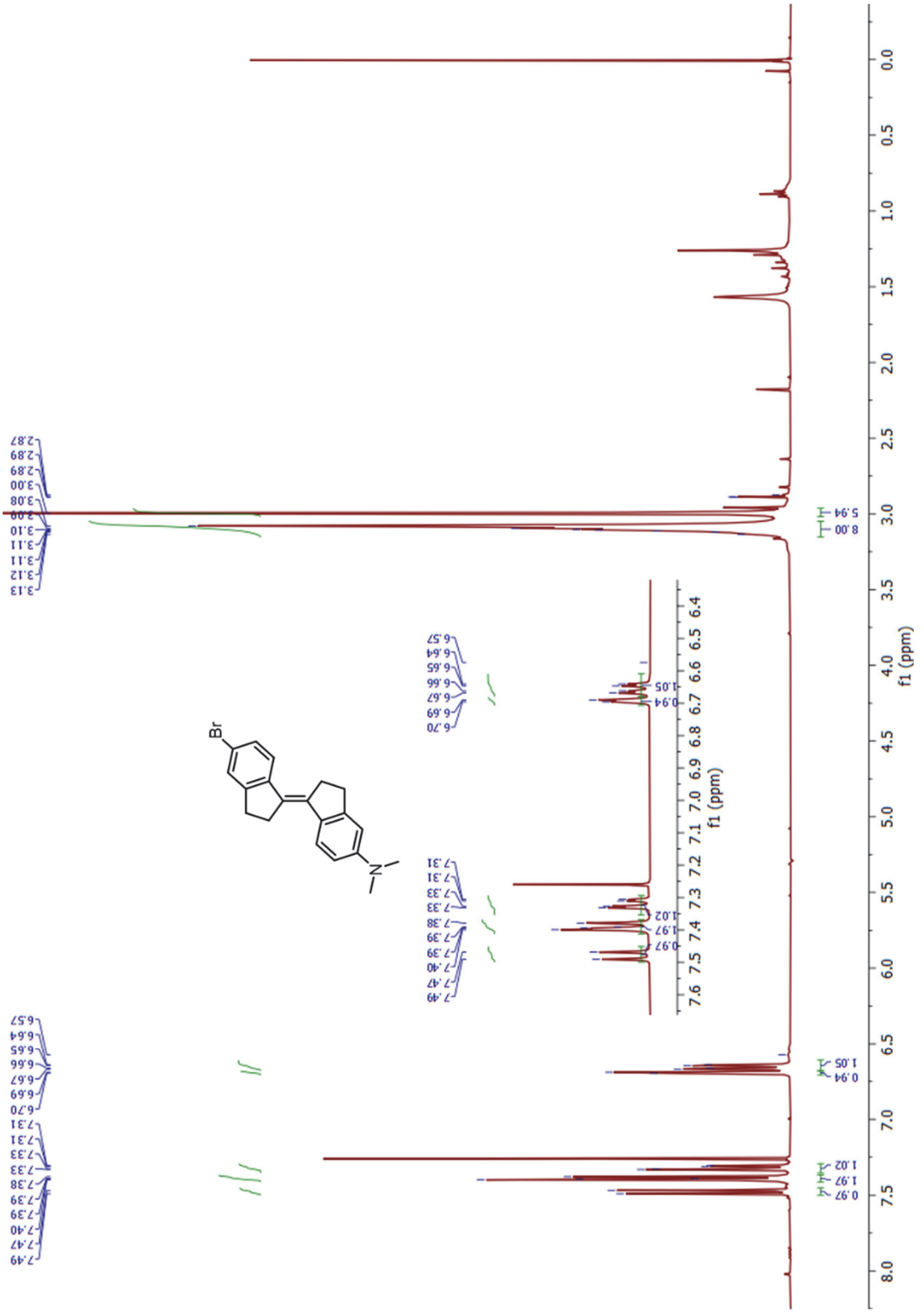

Figure S7. 400 MHz ^{1}H NMR spectrum of (*E*)-**4** measured at 298 K in $CDCl_3$.

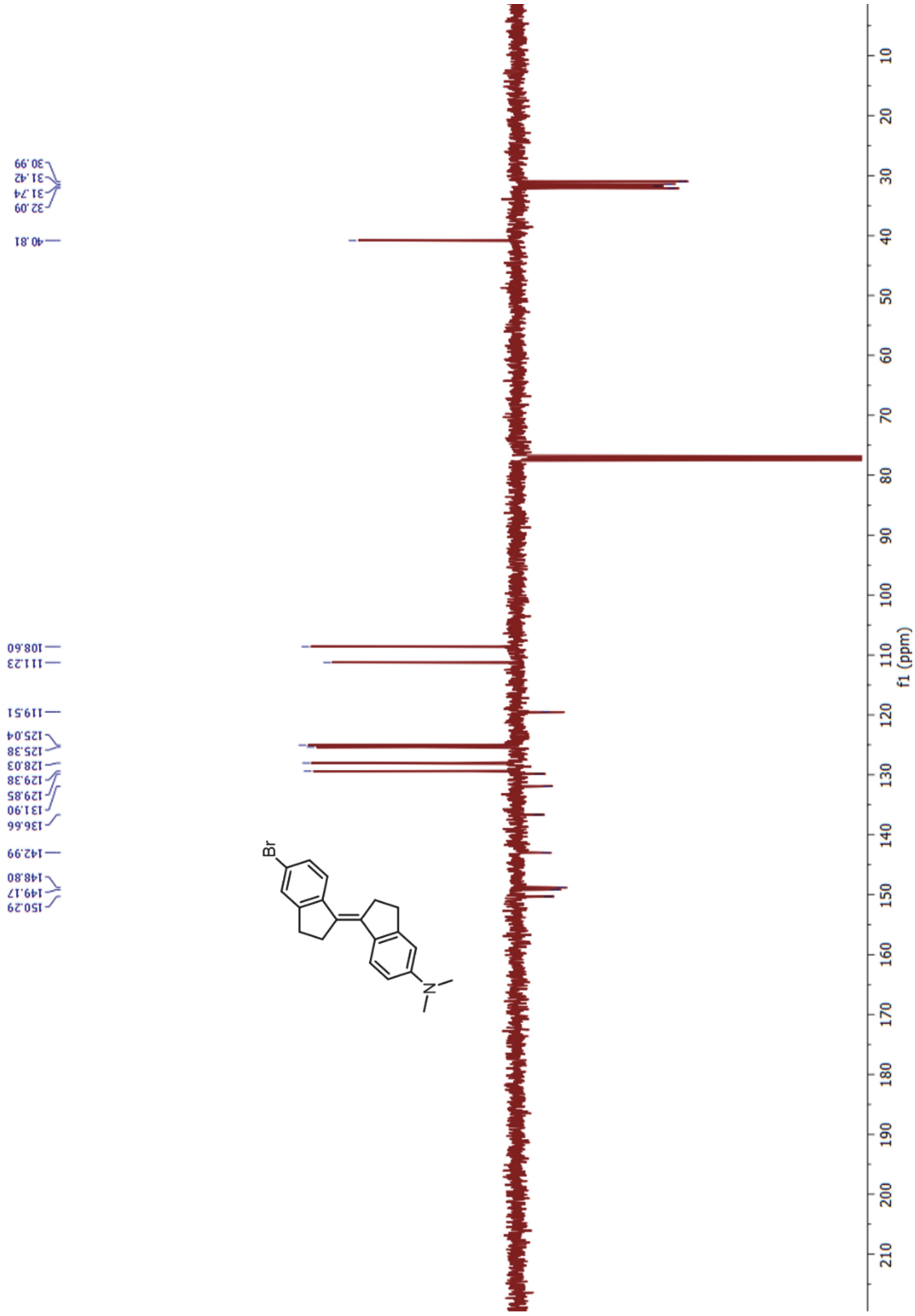

Figure S8. 400 MHz ^{13}C NMR APT spectrum of (*E*)-**4** measured at 298 K in $CDCl_3$: CH and CH_3 signals positive and quaternary carbon and CH_2 signals negative.

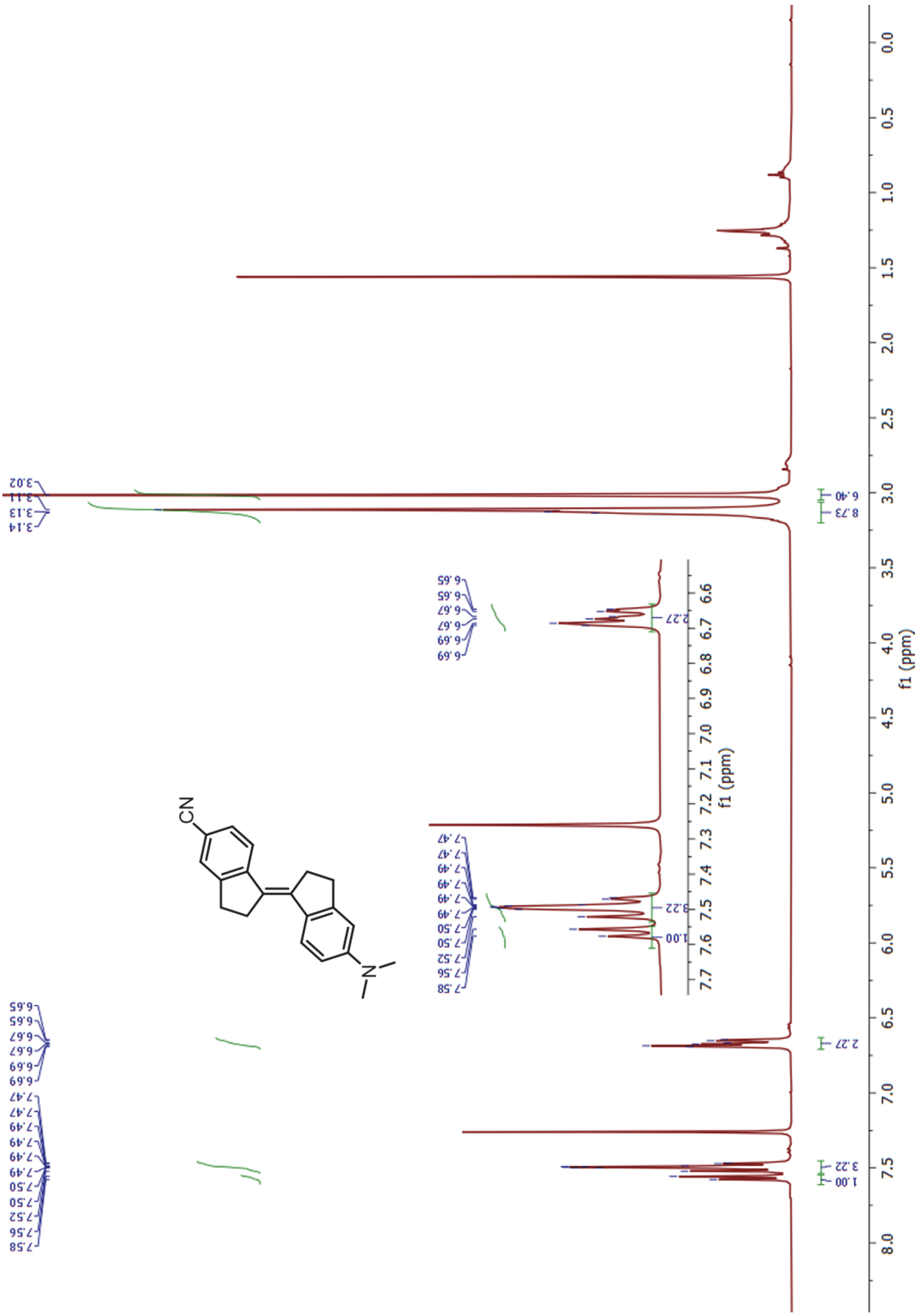

Figure S9. 400 MHz ^{1}H NMR spectrum of (*E*)-**1** measured at 298 K in $CDCl_3$.

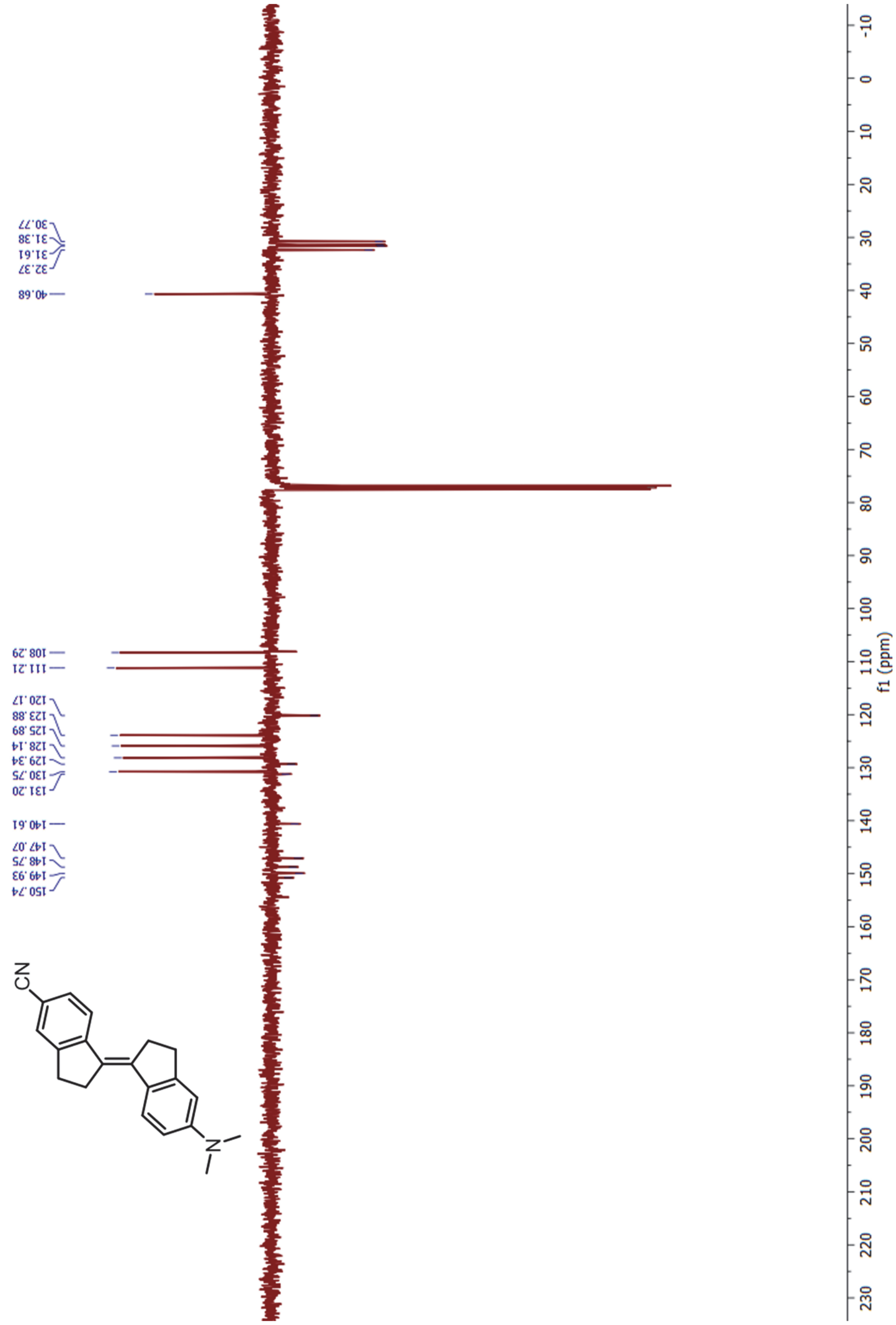

Figure S10. 400 MHz ^{13}C NMR APT spectrum of (*E*)-**1** measured at 298 K in $CDCl_3$: CH and CH_3 signals positive and quaternary carbon and CH_2 signals negative.

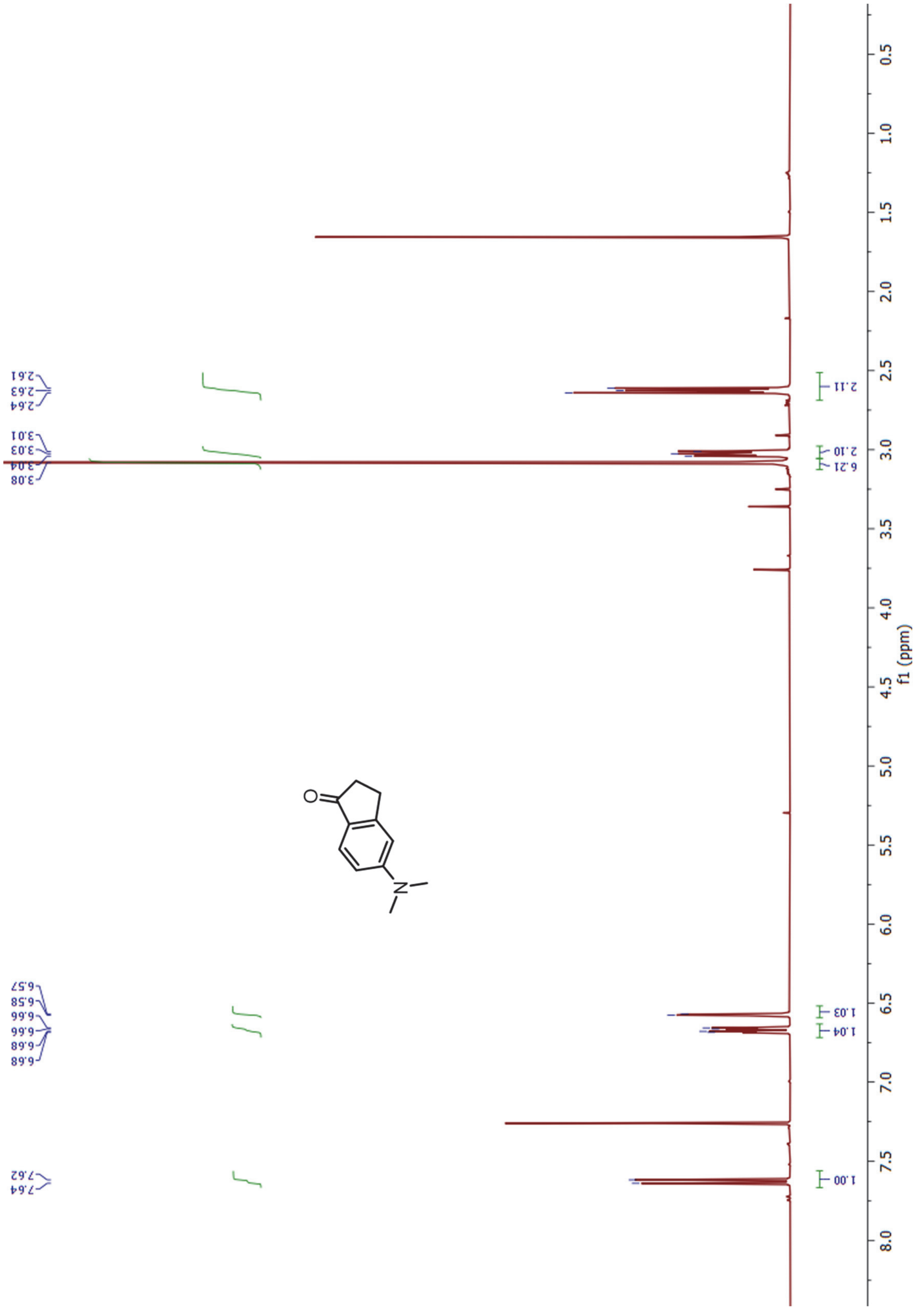

Figure S11. 400 MHz ^{1}H NMR spectrum of **5** measured at 298 K in $CDCl_3$.

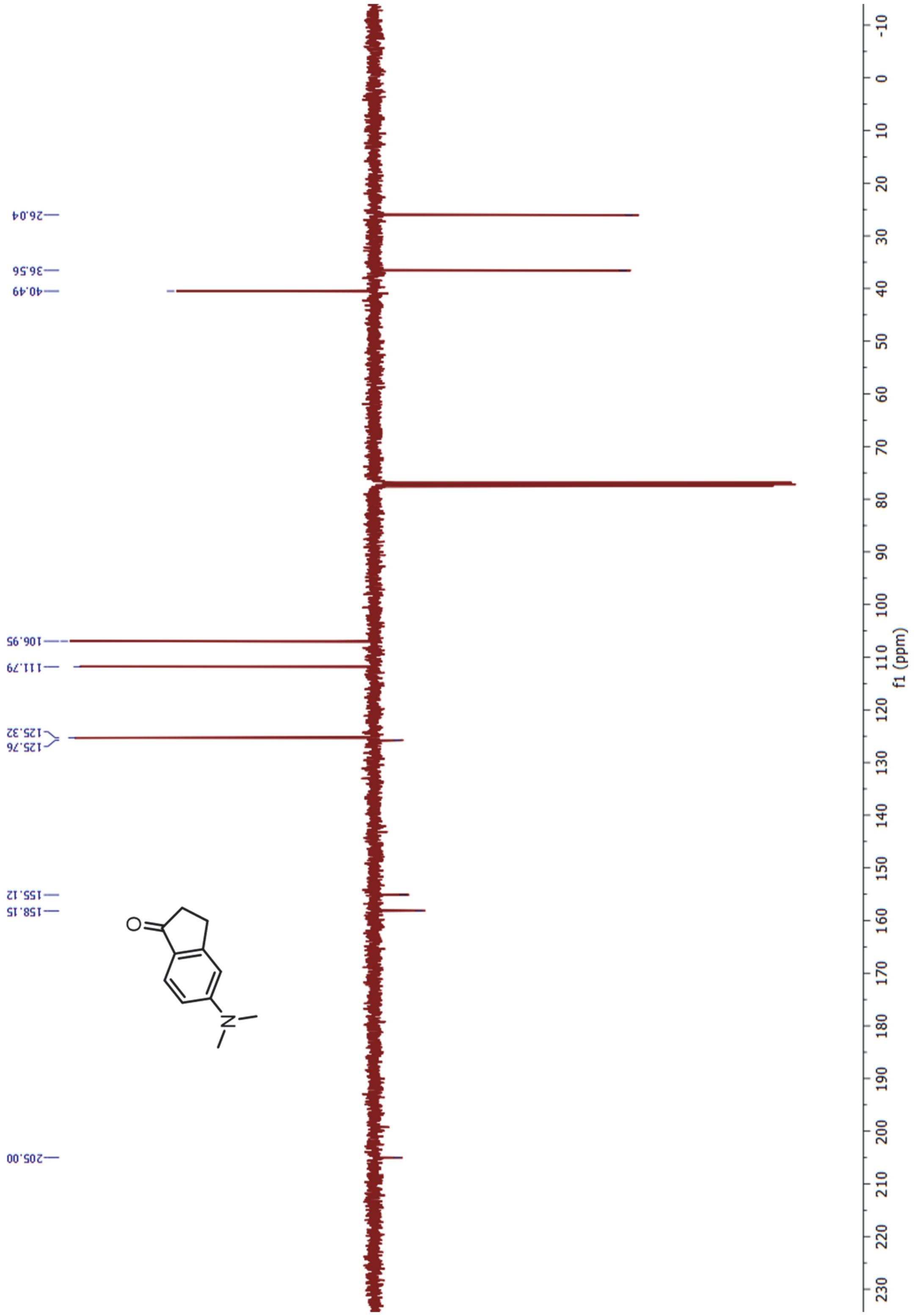

Figure S12. 400 MHz ^{13}C NMR APT spectrum of **5** measured at 298 K in $CDCl_3$: CH and CH_3 signals positive and quaternary carbon and CH_2 signals negative.

^{1}H NMR analysis of mixed McMurry reaction

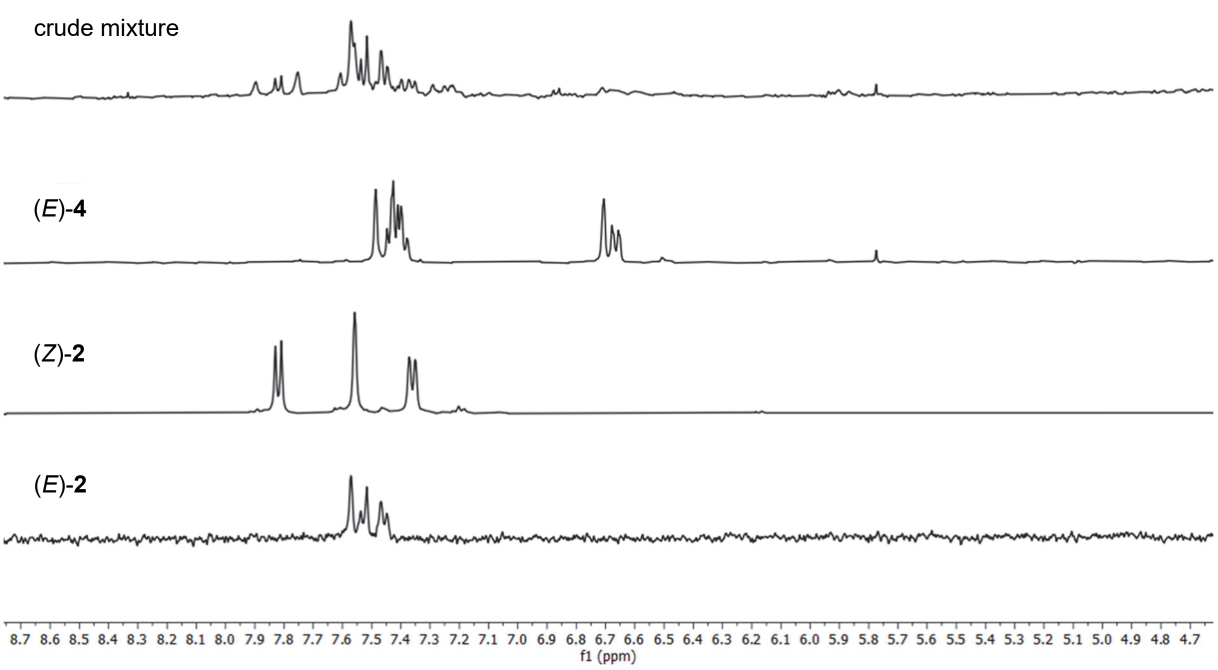

Figure S13. Aromatic region in the ^{1}H NMR spectrum (400 MHz) of the crude mixture obtained after mixed McMurry coupling of 5-bromo-1-indanone and **5** recorded at 298 K in DMSO-d_6. For comparison, the spectra of desymmetrized dimethylamino-bromo substituted (*E*)-**4** and dibromo substituted (*E*)-**2** and (*Z*)-**2** recorded at 298 K in DMSO-d_6 are shown below.

UV-Vis photoisomerization studies

Note: When a sample of (E)-**1** in MeCN was irradiated and a spectrum recorded, the absorption increased upon recording additional spectra. As thermal isomerization is negligible at rt (see Figure S19), this increase was ascribed to $Z\rightarrow E$ back isomerization caused by the deuterium light source (UV range) of the used diode array UV-Vis spectrophotometer. This process could be avoided by placing a longpass filter ($\lambda_{cut\text{-}on}$ = 280±5 nm) in in front of the light source.

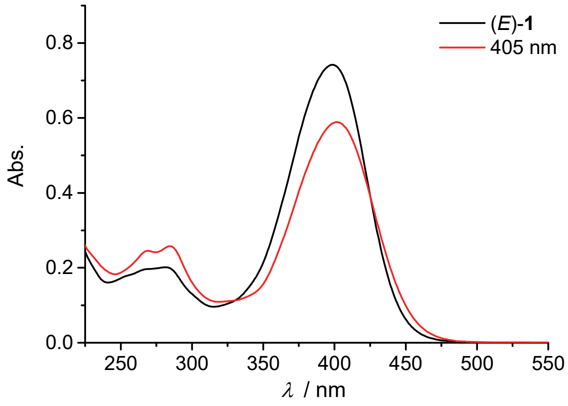

Figure S14. Full UV-Vis absorption spectrum starting with (E)-**1** (2.5×10^{-5} M in degassed MeCN) recorded before and after 405 nm irradiation for 40 s without using an optical filter.

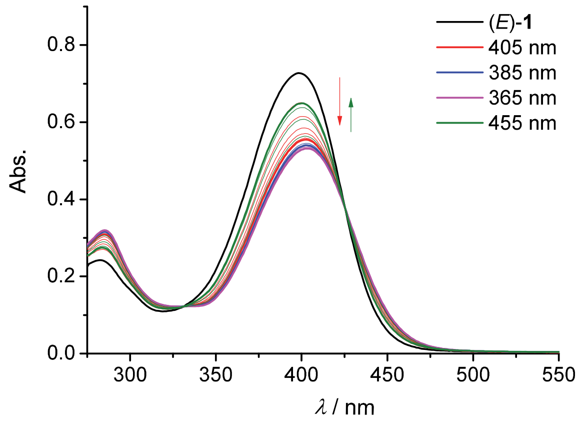

Figure S15. UV-Vis spectral changes of (E)-**1** (2.5×10^{-5} M in degassed MeCN) upon successive 405 nm irradiation (for 2, 4, 8, 12, 16 s), 385 nm irradiation (for 2, 4, 8 s), 365 nm irradiation (for 2, 4, 8 s), and 455 nm irradiation (for 2, 6, 12, 24 s). The spectrum obtained upon 340 nm irradiation (for 8 s) was virtually the same as that using 365 nm, revealing similar PSS ratios. A cut-on filter (λ = 280±5 nm) was placed in front of the light source of the spectrophotometer.

Quantum yield determination

The photon flux of the used Thorlab model M405F1 high-power LED (λ_{max} = 405 nm) was determined at 1/3 of the maximum power by measuring the production of ferrous ions from potassium ferrioxalate.[4] This production was determined at different irradiation times and the slope of a plot of the concentration of Fe^{2+} ions against time (Figure S16) corresponds to the rate (r) of Fe^{2+} ion formation, which was found to be: 1.50×10^{-6} M s^{-1}. Hence, the moles of photons absorbed per time unit ($Nh\nu/t$ = moles of $Fe^{2+}/\phi\, t$) in a 2 mL solution can be calculated using the reported quantum yield of ferrioxalate (ϕ = 1.14),[5] which gives: 2.63×10^{-9} mol s^{-1}.

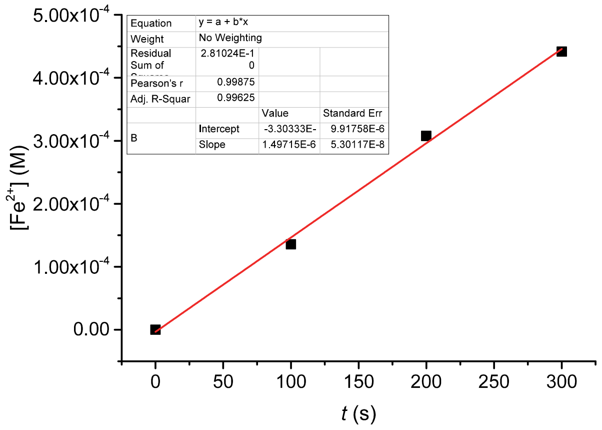

Figure S16. Concentration of Fe^{2+} ions obtained after different 405 nm irradiation periods of a stirred (1200 rpm) 2 mL solution of potassium ferrioxalate (0.15 M) in a 1 cm quartz cuvette.[4]

Next, a solution of (*E*)-**1** in MeCN (1.0×10^{-4} M) in a 1 cm quartz cuvette was irradiated with the same light source in the same cuvette holder and the absorbance increase at λ = 450 nm was monitored over time. The molar absorptivity of (*Z*)-**1** at this wavelength was estimated by deconvolution of the PSS_{405} absorption spectrum using the *E/Z* ratio determined by ^{1}H NMR spectroscopy (see Figure S18) and using the known absorption spectrum of (*E*)-**1**. This deconvolution gives $\varepsilon_{Z(450)} = 5.29 \times 10^3$ M^{-1} cm^{-1} where experimentally determined $\varepsilon_{E(450)}$ = 2.39×10^3 M^{-1} cm^{-1}. These molar absorptivities were used to the calculate the production of (*Z*)-isomer according to Δ[(*Z*)-**1**] = ΔAbs. / ($\varepsilon_Z - \varepsilon_E$). The slope of the plots of concentration increase against time obtained from two runs (Figure S17), at initial and low conversion, corresponds to the rate of (Z)-**1** formation. The photochemical quantum yield was obtained by comparison of this rate of formation of (*Z*)-**1** ($r = 3.57 \pm 0.03 \times 10^{-7}$ M s^{-1}) with the rate of Fe^{2+} formation from potassium ferrioxalate giving $\Phi_{Z\rightarrow E} = 27.2 \pm 0.26\%$. The quantum yield for the

'reverse' photoisomerization process is defined as: $\Phi_{Z\rightarrow E} = \Phi_{E\rightarrow Z}\, \varepsilon_E\, n_E / \varepsilon_Z\, n_Z$, where ε_E and ε_Z are the molar absorptivities at the irradiation wavelength $\lambda = 405$ nm [$\varepsilon_{Z(405)} = 1.78 \times 10^4$ M^{-1} cm^{-1} and $\varepsilon_{E(405)} = 2.83 \times 10^4$ M^{-1} cm^{-1}] and n_E and n_Z are the amount of (*E*)-**1** and (*Z*)-**1** at the photostationary state [PSS$_{405}$ (*E*/*Z*) = 41:59], giving $\Phi_{Z\rightarrow E} = 29.9 \pm 0.29\%$.

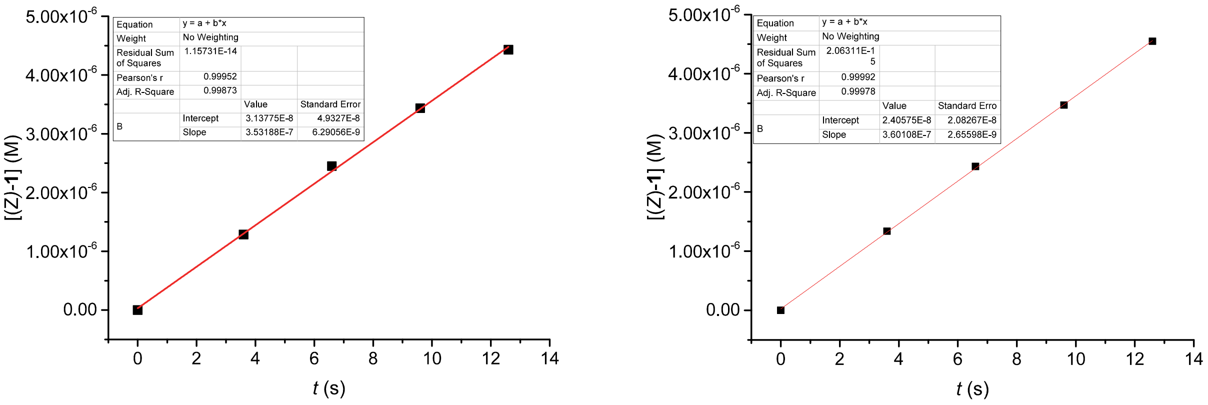

Figure S17. Two kinetic runs showing the production of (*Z*)-**1** as a function of time during 405 nm irradiation of stirred (1200 rpm) solutions of (*E*)-**1** (1.0×10^{-4} M in MeCN) in a 1 cm quartz cuvette (Abs$_{405}$ > 2): $r_1 = 3.53 \times 10^{-7}$ M s^{-1}, $r_2 = 3.60 \times 10^{-7}$ M s^{-1}. The concentration of (*Z*)-**1** was determined by using the absorbance increase at $\lambda = 450$ nm and a cut-on filter ($\lambda = 360\pm5$ nm) was placed in front of the light beam of the spectrophotometer.

^{1}H NMR photoisomerization studies

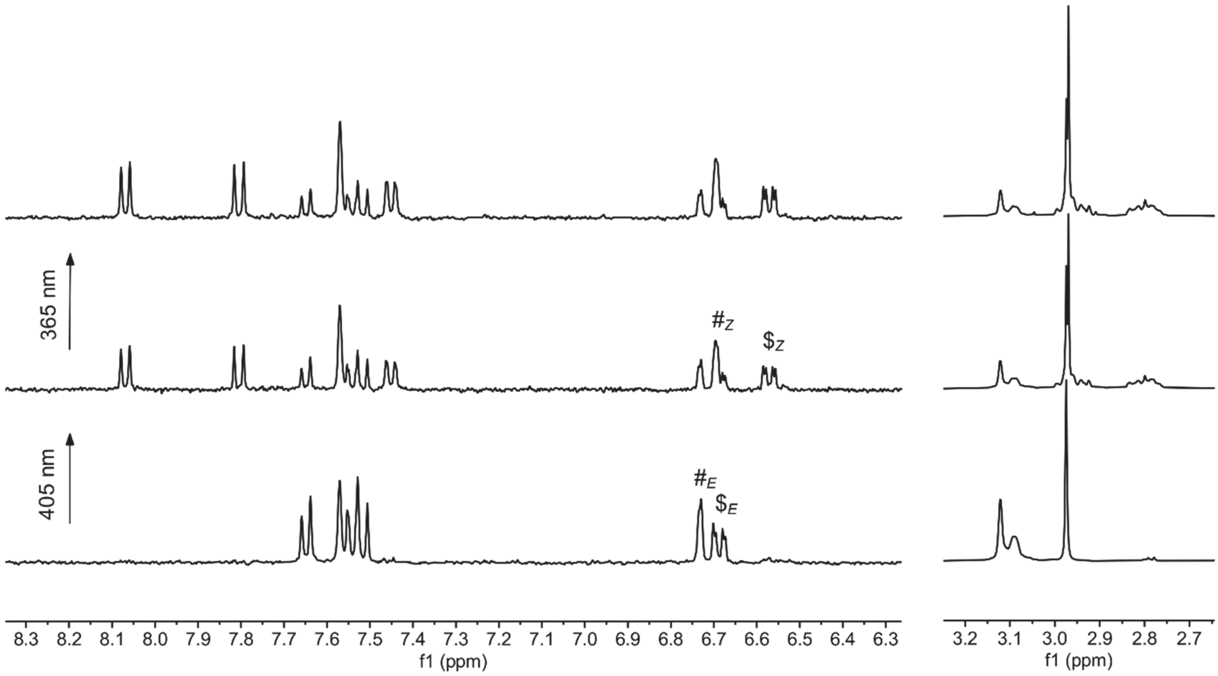

Figure S18. Aromatic and aliphatic region in the ^{1}H NMR spectrum (400 MHz, 293 K) of (*E*)-**1** (2.8 mM in degassed MeCN-d_3) before (bottom) and after consecutive irradiation with 405 nm light for 30 min (middle) and 365 nm light for 45 min (top). PSS_{405} and PSS_{365} (*E/Z*) ratios were determined by integration of the indicated signals (#,$) as 41:59 and 34:66, respectively.

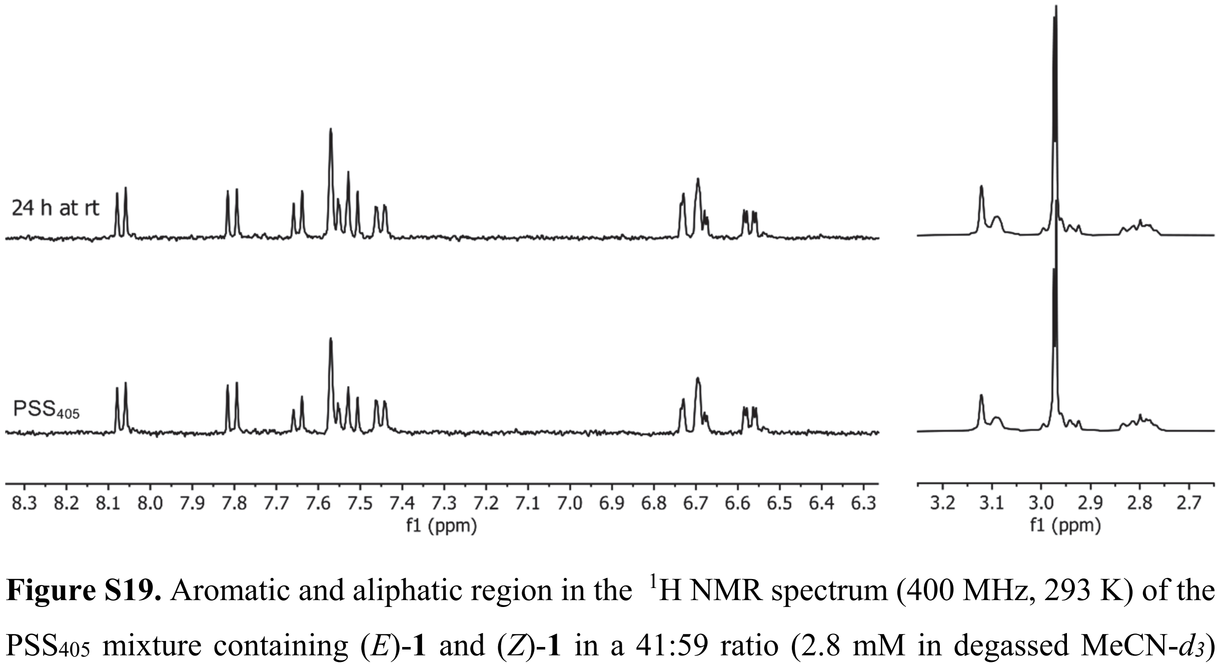

Figure S19. Aromatic and aliphatic region in the ^{1}H NMR spectrum (400 MHz, 293 K) of the PSS_{405} mixture containing (*E*)-**1** and (*Z*)-**1** in a 41:59 ratio (2.8 mM in degassed MeCN-d_3) before (bottom) and after standing for 24 h in the dark (top). The (*E/Z*) ratio in the top spectrum was determined as 47:53, which would suggest some (very slow) back isomerization.

Table S1. PSS ratios estimated using UV-Vis absorption and ^{1}H NMR (PSS_{365}) data.[a]

	Abs. (400 nm)	Δ Abs. (400 nm)	x_z (%)	PSS (*E/Z*)
(*E*)-**1**	0.73	-	0	-
PSS_{455}	0.65	0.08	26	74:26
PSS_{405}	0.55	0.17	58	42:58[b]
PSS_{385}	0.54	0.19	63	37:63
**PSS_{365}**	**0.53**	**0.20**	**66**	**34:66**

[a] Calculated using the PSS_{365} value determined by ^{1}H NMR spectroscopy (see Figure S18) and the absorption at $\lambda = 400$ nm, mole fraction (*Z*)-**1** (x_z) = [Δ Abs. / Δ Abs. (PSS_{365})] × x_z (PSS_{365}).
[b] By ^{1}H NMR integration determined as 41:59 (*E/Z*) and thus within ±5% integration error.

UV-Vis studies in the presence of acid

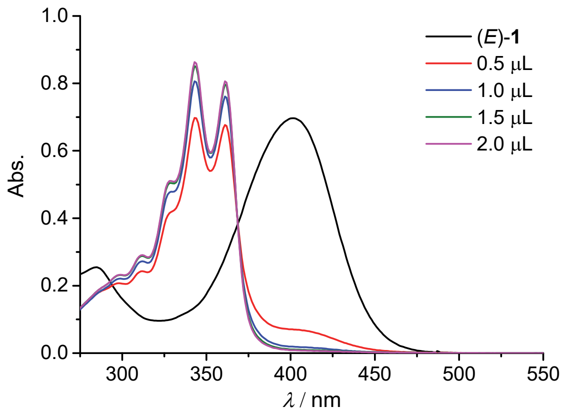

Figure S20. UV-Vis spectral changes of (*E*)-**1** (2.5×10^{-5} M in MeCN) recorded at 253 K upon stepwise addition of a 10× diluted solution of TFA in MeCN (1.3 M). Compound (*E*)-**1** is fully protonated in the presence of 2 μL of this solution, corresponding to 0.2 μL TFA.

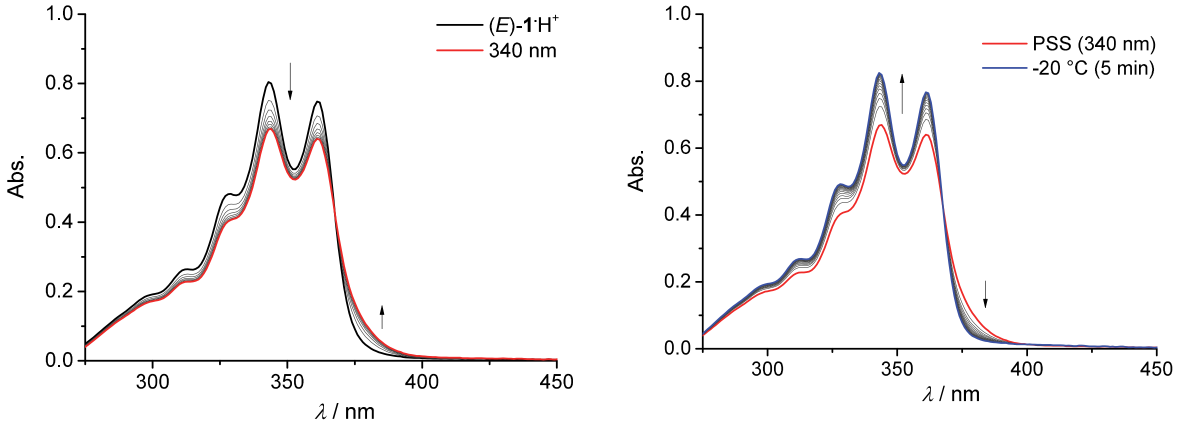

Figure S21. (left) UV-Vis spectral changes of (*E*)-**1** (2.5×10^{-5} M in MeCN) recorded at 253 K in the presence of 1 μL TFA (0.05% v/v) upon irradiation with 340 nm light for 3 min in total and (right) upon standing in the dark for a total of 5 min (spectra recorded with 20 s intervals).

For the time-based measurements, 1 μL of TFA was added to 2 mL of a stirred (1200 rpm) solution of (*E*)-**1** (2.5×10^{-5} M in MeCN) at −20 °C. The solution was irradiated with 340 nm light for 3 min and thereafter a UV-Vis spectrum was recorded every 5 s. A cut-on filter (λ = 360±5 nm) was placed in front of the light source of the spectrophotometer to minimize undesired photoisomerization. The rate constants (k) were obtained by fitting to the equation $A = A_0e^{-kt}$ using OriginPro 9.1 software. The average of this constant was used to calculate half-life ($t_{1/2}$) and Gibbs free energy of activation ($\Delta^{\ddagger}G°$) using the following equations:

$$t_{1/2} = \frac{\ln(2)}{k}$$

$$\Delta^{\ddagger}G = RT[\ln\left(\frac{k_{\mathrm{B}}}{h}\right) - \ln\left(\frac{k}{\mathrm{T}}\right)]$$

Where k_{B} is the Boltzmann constant, h the Planck constant, and R the Gas constant.

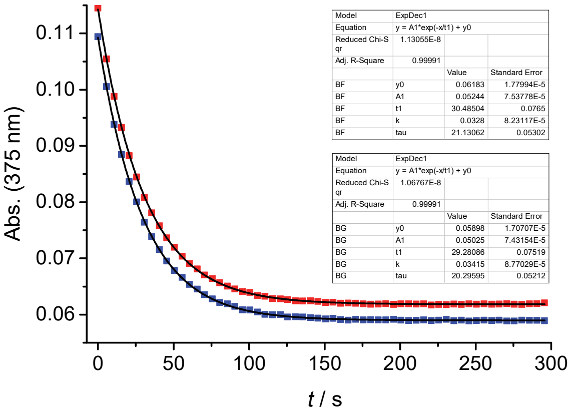

Figure S22. Change of the 375 nm absorption in time at 253 K of solutions of (*E*)-**1** (2.5×10^{-5} M in MeCN) that were irradiated at 340 nm for 3 min: $k_1 = 3.28 \times 10^{-2}$ s^{-1}, $k_2 = 3.42 \times 10^{-2}$ s^{-1}.

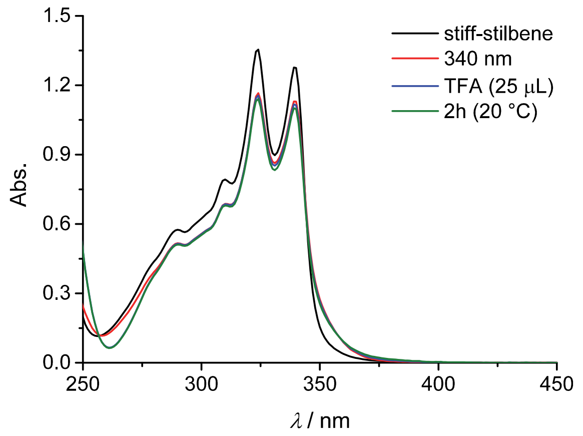

Figure S23. UV-Vis spectral changes of stiff-stilbene (*E*)-**6** (3.3×10^{-5} M in MeCN) upon 340 nm irradiation for 5 min, TFA (25 µL, 1.25% v/v) addition, and standing for 2 h at rt.

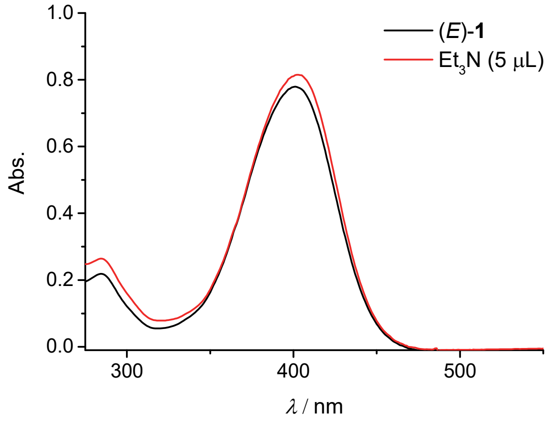

Figure S24. UV-Vis spectra of (*E*)-**1** (2.5×10^{-5} M in MeCN) at 253 K in absence and presence of Et_3N (5 µL, 0.25% v/v). The change is similar as in the acid/base cycle shown in Figure 3.

^{1}H NMR studies in the presence of acid

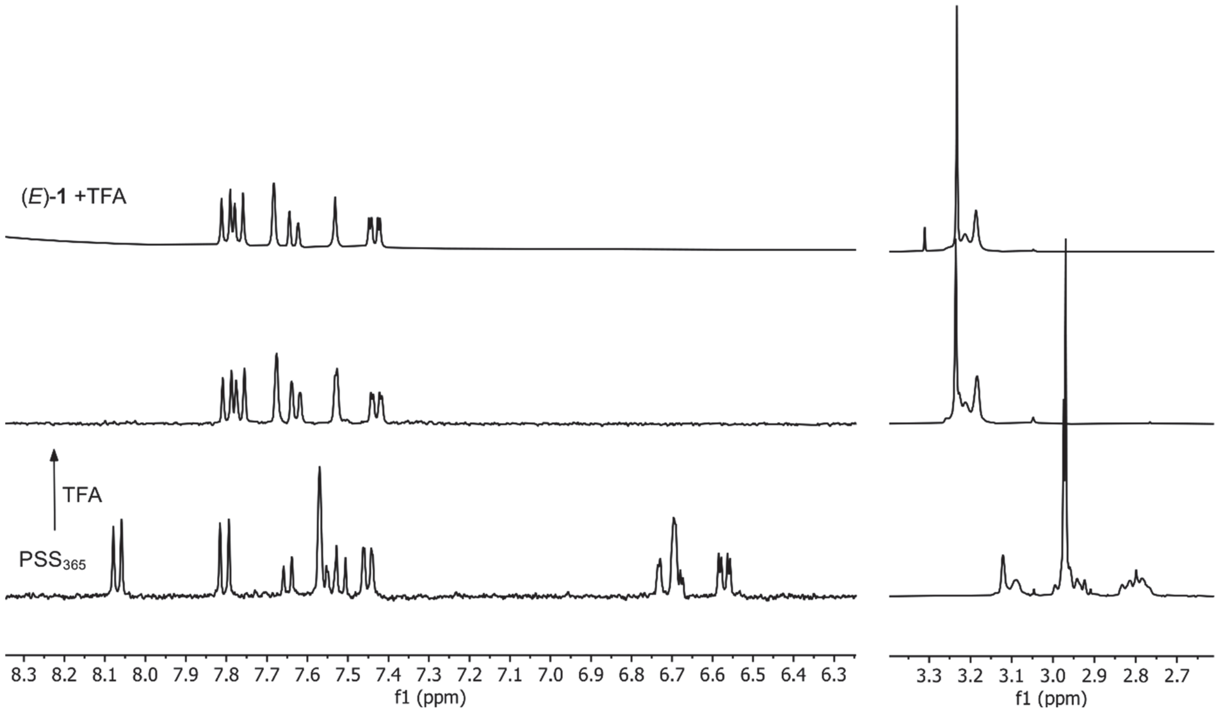

Figure S25. Aromatic and aliphatic region in the ^{1}H NMR spectrum (400 MHz, 293 K) of the PSS_{365} mixture containing (*E*)-**1** and (*Z*)-**1** in a 34:66 (*E/Z*) ratio (2.8 mM in degassed MeCN-d_3) before (bottom) and 10 min. after addition of one drop of TFA (middle), and a separate sample of (*E*)-**1** with one drop of TFA (top).

Geometry optimizations by DFT

Calculations were performed using Gaussian 09[6] and data was visualized using Avogadro 1.2.0.[7] Geometry optimizations were performed at the B3LYP/6-31G(d,p) level of theory using and IEFPCM acetonitrile solvation model. All computed stationary points were subjected to frequency analysis and confirmed as local minima.

Table S2. Cartesian coordinates of (*E*)-**1**.

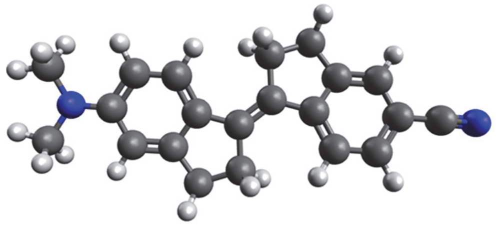

Atom	X	Y	Z
C	3.27383	1.15614	-0.18638
C	2.46370	0.01988	0.06190
C	2.42337	2.37645	-0.44435
C	0.99206	1.92313	-0.06190
C	1.04833	0.39877	0.02885
C	4.65505	1.07121	-0.19415
C	5.26740	-0.17407	0.06069
C	4.47569	-1.30811	0.32223
C	3.08835	-1.21536	0.32507
C	-3.64775	-1.11553	-0.18104
C	-4.29947	0.13148	0.00294
C	-3.48272	1.27226	0.23076
C	-2.09714	1.18505	0.24751
C	-1.45276	-0.04960	0.04298
C	-2.26488	-1.18739	-0.15865
C	-1.41632	-2.42131	-0.34907
C	-0.03747	-0.42577	0.03979
C	0.01531	-1.95217	0.01043
N	-5.67356	0.23406	-0.03533
C	-6.48905	-0.96785	-0.13617
C	-6.32119	1.49749	0.28866
C	6.69248	-0.28350	0.06278
N	7.85472	-0.37170	0.06312
H	2.47690	2.65721	-1.50373
H	2.75887	3.24664	0.12810
H	0.25079	2.26151	-0.79154
H	0.70481	2.35875	0.90430
H	5.26763	1.94586	-0.38954
H	2.51179	-2.10101	0.55399
H	-1.53262	2.08686	0.44825
H	-4.22314	-2.02076	-0.33396
H	0.30230	-2.34023	0.99643
H	0.75744	-2.32300	-0.70213
H	-1.46571	-2.76302	-1.39076

H	-1.75263	-3.25939	0.26998
H	4.95569	-2.25827	0.53120
H	-3.93949	2.23769	0.40776
H	-7.54033	-0.68354	-0.17488
H	-6.34751	-1.64321	0.71956
H	-6.26085	-1.52808	-1.05027
H	-7.39876	1.38552	0.17139
H	-5.99399	2.29672	-0.38605
H	-6.12011	1.81998	1.32011

Sum of electronic and zero-point Energies = -921.457654
Sum of electronic and thermal Energies = -921.437534
Sum of electronic and thermal Enthalpies = -921.436590
Sum of electronic and thermal Free Energies = -921.507405

Table S3. Cartesian coordinates of (*Z*)-**1**.

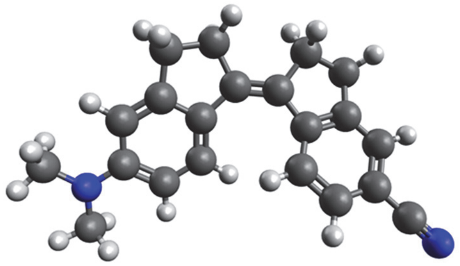

Atom	X	Y	Z
C	-3.44523	0.74792	0.34235
C	-2.13056	0.38125	-0.03839
C	-3.48897	2.19642	0.76839
C	-2.15781	2.76722	0.22143
C	-1.24587	1.54898	0.05294
C	-4.49003	-0.15905	0.29115
C	-4.23962	-1.46706	-0.17379
C	-2.94567	-1.83137	-0.59382
C	-1.90125	-0.91632	-0.53052
C	3.61432	0.58286	-0.32170
C	3.64426	-0.78444	0.05543
C	2.42039	-1.37745	0.47011
C	1.22718	-0.66812	0.45643
C	1.19081	0.67033	0.02954
C	2.41585	1.27840	-0.32270
C	2.20633	2.73681	-0.65619
C	0.10984	1.66174	-0.00405
C	0.78756	3.02825	-0.10989
N	4.81714	-1.51001	0.03372
C	4.85317	-2.85433	0.59305
C	6.07842	-0.83934	-0.24817
C	-5.30454	-2.41834	-0.23936
N	-6.17373	-3.19306	-0.29184
H	-3.51743	2.26717	1.86366
H	-4.37120	2.72183	0.39150
H	-1.72868	3.52453	0.88279
H	-2.33211	3.25310	-0.74915
H	-5.49357	0.12605	0.59121
H	-0.92363	-1.20903	-0.89151
H	0.33288	-1.16072	0.81970

H	4.52707	1.09956	-0.59320
H	0.85620	3.48811	0.88591
H	0.22728	3.72370	-0.74081
H	2.23831	2.89123	-1.74277
H	2.97523	3.38358	-0.22226
H	-2.77033	-2.82996	-0.97971
H	2.40650	-2.40089	0.82276
H	5.85000	-3.27165	0.45195
H	4.14137	-3.51388	0.08453
H	4.62360	-2.86996	1.66817
H	6.87827	-1.57929	-0.27090
H	6.33207	-0.08189	0.50761
H	6.05283	-0.34627	-1.22618

Sum of electronic and zero-point Energies = -921.452643
Sum of electronic and thermal Energies = -921.432605
Sum of electronic and thermal Enthalpies = -921.431661
Sum of electronic and thermal Free Energies = -921.50190

Table S4. Cartesian coordinates of (*E*)-**1**·H$^+$.

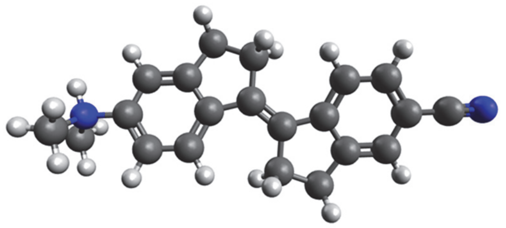

Atom	X	Y	Z
C	-3.48567	-1.11382	0.25484
C	-4.21300	0.05003	0.01242
C	-3.59055	1.27597	-0.21346
C	-2.20153	1.32205	-0.19615
C	-1.43363	0.16105	0.03871
C	-2.09291	-1.05821	0.27208
C	-1.32149	2.52363	-0.42233
C	0.09545	2.02312	-0.04855
C	-0.00039	0.50091	0.02636
C	2.37301	-2.39437	-0.34258
C	0.95348	-1.88379	0.00692
C	1.05126	-0.36035	0.04392
C	2.48296	-0.02063	0.06369
C	3.25449	-1.18785	-0.14059
C	3.13689	1.20608	0.28015
C	4.52640	1.26038	0.27259
C	5.28232	0.09399	0.05265
C	4.63978	-1.14193	-0.15287
N	-5.69948	0.00299	-0.00408
C	-6.24374	-0.81141	-1.15366
C	-6.28857	-0.42856	1.31823
C	6.71212	0.16387	0.04814
N	7.87545	0.21988	0.04333
H	-6.01641	0.96452	-0.15876
H	-3.98096	-2.06047	0.43936
H	-4.17262	2.17549	-0.39477
H	-1.54772	-1.96567	0.48889

H	-1.63597	3.38635	0.17229
H	-1.36676	2.83175	-1.47401
H	0.39400	2.43448	0.92386
H	0.84332	2.34931	-0.77584
H	2.42421	-2.73207	-1.38477
H	2.68085	-3.24106	0.27809
H	0.64547	-2.26888	0.98732
H	0.21142	-2.22784	-0.71832
H	2.58547	2.11493	0.47592
H	5.03390	2.20323	0.44437
H	5.22771	-2.03980	-0.31315
H	-5.81311	-0.42683	-2.07634
H	-7.32810	-0.70750	-1.16205
H	-5.96319	-1.85239	-1.00617
H	-5.89968	0.22778	2.09446
H	-6.00113	-1.46013	1.51018
H	-7.37254	-0.34625	1.25152

Sum of electronic and zero-point Energies = -921.889565
Sum of electronic and thermal Energies = -921.869780
Sum of electronic and thermal Enthalpies = -921.868836
Sum of electronic and thermal Free Energies = -921.938478

Table S5. Cartesian coordinates of (*Z*)-**1**·H^+.

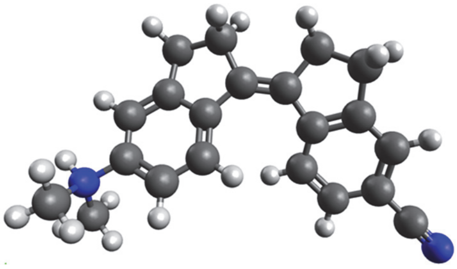

Atom	X	Y	Z
C	-2.44861	-1.23265	0.47239
C	-3.60351	-0.58375	0.03668
C	-3.59111	0.74414	-0.38446
C	-2.37707	1.42222	-0.38596
C	-1.18209	0.77903	0.00174
C	-1.23605	-0.54623	0.45984
C	-2.12159	2.86349	-0.74878
C	-0.70970	3.12682	-0.17032
C	-0.06864	1.74530	-0.04467
N	6.02044	-3.35467	-0.15887
C	5.18320	-2.54527	-0.14026
C	4.44731	-0.24977	0.33776
C	4.15432	-1.55032	-0.11535
C	2.86036	-1.87225	-0.56738
C	1.85179	-0.91573	-0.54293
C	3.43645	0.69926	0.35056
N	-4.90193	-1.30820	0.03809
C	-4.91784	-2.47999	-0.91496
C	-5.35102	-1.70139	1.42544
C	2.12190	0.37338	-0.05548
C	1.27699	1.57904	0.00866
C	2.23251	2.76189	0.16074

C	3.53077	2.15330	0.74433
H	-5.59914	-0.64571	-0.31300
H	-2.47766	-2.25493	0.83300
H	-4.50598	1.24501	-0.68968
H	-0.35312	-1.04534	0.83700
H	-2.88403	3.53889	-0.35108
H	-2.12164	2.98513	-1.83945
H	-0.79414	3.59398	0.82042
H	-0.12182	3.80348	-0.79476
H	5.45409	0.00027	0.65630
H	2.65773	-2.86752	-0.94734
H	0.87548	-1.17330	-0.93327
H	-4.63891	-2.11672	-1.90244
H	-5.92447	-2.89547	-0.92744
H	-4.20497	-3.22455	-0.56680
H	-5.36146	-0.80678	2.04505
H	-4.65181	-2.43302	1.82520
H	-6.34922	-2.13115	1.35310
H	1.82036	3.55282	0.79165
H	2.43819	3.20759	-0.82219
H	4.43831	2.63342	0.36824
H	3.54431	2.25091	1.83730

Sum of electronic and zero-point Energies = -921.884722
Sum of electronic and thermal Energies = -921.864858
Sum of electronic and thermal Enthalpies = -921.863914
Sum of electronic and thermal Free Energies = -921.933607

Table S6. Cartesian coordinates of (*E*)-stiff-stilbene.

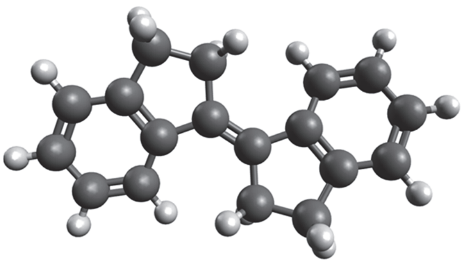

Atom	X	Y	Z
C	-2.84785	0.94792	0.18236
C	-1.96218	-0.11966	-0.08385
C	-2.08897	2.22952	0.42842
C	-0.63975	1.89278	-0.00251
C	-0.57025	0.36844	-0.06896
C	-4.22497	0.75036	0.20847
C	-4.74024	-0.52528	-0.04438
C	-3.87429	-1.58699	-0.32480
C	-2.49123	-1.39519	-0.34816
C	4.22497	-0.75036	0.20847
C	4.74024	0.52528	-0.04438
C	3.87429	1.58699	-0.32480
C	2.49123	1.39519	-0.34816
C	1.96218	0.11966	-0.08385
C	2.84785	-0.94792	0.18236
C	2.08897	-2.22952	0.42842

C	0.57025	-0.36844	-0.06896
C	0.63975	-1.89278	-0.00252
H	-2.12713	2.49797	1.49222
H	-2.50728	3.07766	-0.12300
H	0.10014	2.30874	0.68664
H	-0.43291	2.32770	-0.98944
H	-4.89513	1.58044	0.41688
H	-1.84805	-2.23025	-0.59444
H	1.84804	2.23025	-0.59445
H	4.89513	-1.58044	0.41688
H	0.43291	-2.32770	-0.98944
H	-0.10014	-2.30874	0.68664
H	2.12713	-2.49797	1.49222
H	2.50728	-3.07766	-0.12300
H	-4.27990	-2.57232	-0.53588
H	4.27989	2.57232	-0.53588
H	-5.81383	-0.68932	-0.03245
H	5.81383	0.68933	-0.03245

Sum of electronic and zero-point Energies = -695.303549
Sum of electronic and thermal Energies = -695.289939
Sum of electronic and thermal Enthalpies = -695.288995
Sum of electronic and thermal Free Energies = -695.344266

Table S7. Cartesian coordinates of (*Z*)-stiff-stilbene.

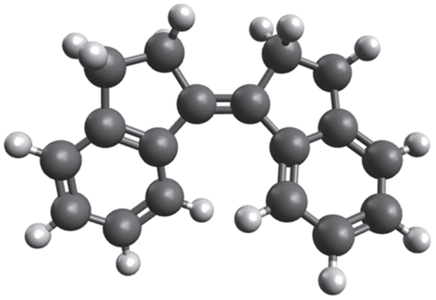

Atom	X	Y	Z
C	2.94908	0.38867	-0.23402
C	1.66846	-0.10274	0.10229
C	2.87736	1.84830	-0.61686
C	1.48687	2.28384	-0.09341
C	0.67813	0.98917	0.00455
C	4.07225	-0.43148	-0.17126
C	3.93143	-1.75540	0.25668
C	2.67386	-2.23987	0.63285
C	1.54388	-1.42317	0.56235
C	-4.07225	-0.43148	0.17126
C	-3.93143	-1.75540	-0.25668
C	-2.67386	-2.23987	-0.63285
C	-1.54388	-1.42317	-0.56235
C	-1.66846	-0.10274	-0.10229
C	-2.94908	0.38867	0.23402
C	-2.87736	1.84830	0.61686
C	-0.67813	0.98917	-0.00455
C	-1.48687	2.28384	0.09341
H	2.93022	1.96281	-1.70791

H	3.69845	2.43701	-0.19638
H	1.01082	3.02783	-0.73734
H	1.59130	2.74102	0.90081
H	5.05182	-0.04263	-0.43718
H	0.58629	-1.80839	0.89129
H	-0.58629	-1.80839	-0.89130
H	-5.05182	-0.04263	0.43718
H	-1.59130	2.74102	-0.90081
H	-1.01082	3.02783	0.73734
H	-2.93022	1.96281	1.70791
H	-3.69845	2.43701	0.19638
H	2.57471	-3.25918	0.99494
H	-2.57471	-3.25918	-0.99494
H	-4.80178	-2.40242	-0.31566
H	4.80178	-2.40242	0.31566

Sum of electronic and zero-point Energies = -695.298686
Sum of electronic and thermal Energies = -695.285057
Sum of electronic and thermal Enthalpies = -695.284113
Sum of electronic and thermal Free Energies = -695.339391

Time-dependent DFT calculations

Using the DFT geometry-optimized structures, TD-DFT calculations (solving for 30 singlet excited states) were performed at the same B3LYP/6-31G(d,p) level of theory and IEFPCM acetonitrile solvation model using Gaussian 09.[6] The output data was again visualized using Avogadro 1.2.0.[7]

(*E*)-1	Symmetry	eV	nm	Oscillator strength	<S²>	Wavefunctions	coefficients
1	A	2.90	427.54 nm	1.1551	0.00	80 → 81	0.70657

(*Z*)-1	Symmetry	eV	nm	Oscillator strength	<S²>	Wavefunctions	coefficients
1	A	2.80	442.57 nm	0.7102	0.00	80 → 81	0.70623

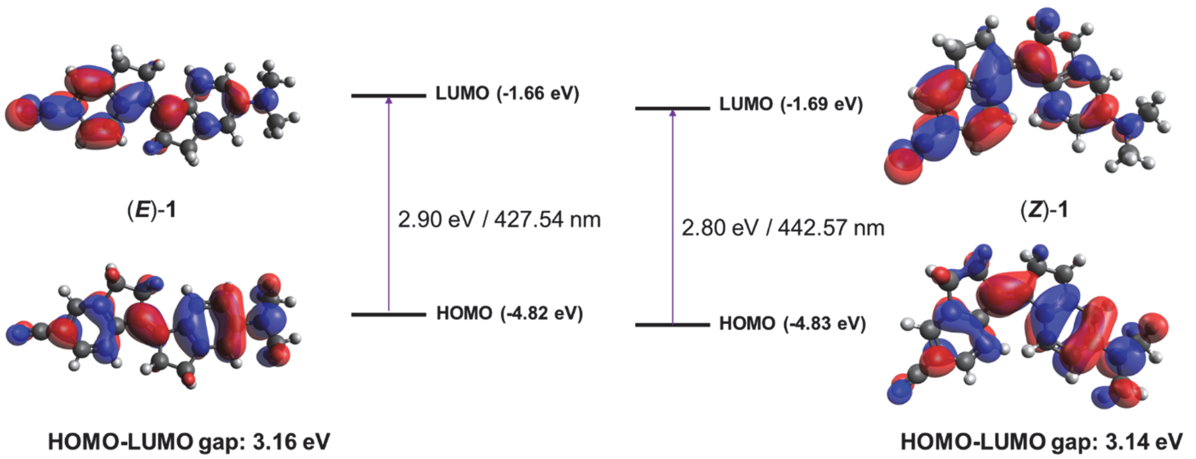

Figure S26. Description and visualization of first excited state of (*E*)-**1** and (*Z*)-**1** described by an excitation from HOMO (wavefunction 80) to LUMO (wavefunction 81). Molecular orbitals are visualized with an isosurface value of 0.02000. The $S_0 \rightarrow S_1$ excitation energies are listed alongside the arrows, representing the optical gaps. The HOMO-LUMO gaps are listed below.

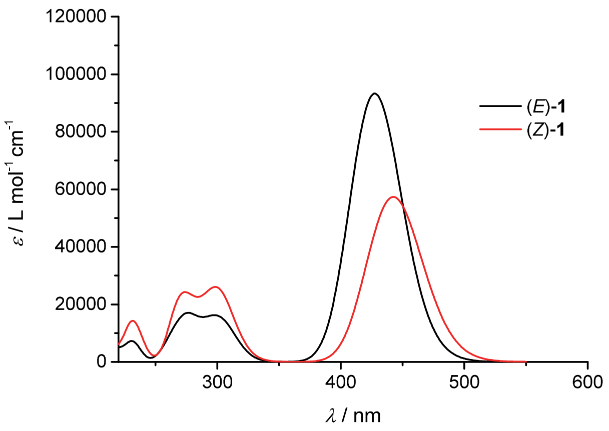

Figure S27. (A) Computed UV-Vis absorption spectra [B3LYP 6-31G(d,p) PCM (CH3CN)] plotted with peak half-width at half height set to 0.167 eV of (*E*)-**1** and (*Z*)-**1**.

(*E*)-1·H+	Symmetry	eV	nm	Oscillator strength	<S²>	Wavefunctions	coefficients
1	A	3.42	362.56 nm	1.1809	0.00	80 → 81	0.70595

(*Z*)-1·H+	Symmetry	eV	nm	Oscillator strength	<S²>	Wavefunctions	coefficients
1	A	3.34	371.51 nm	0.7948	0.00	80 → 81	0.70513

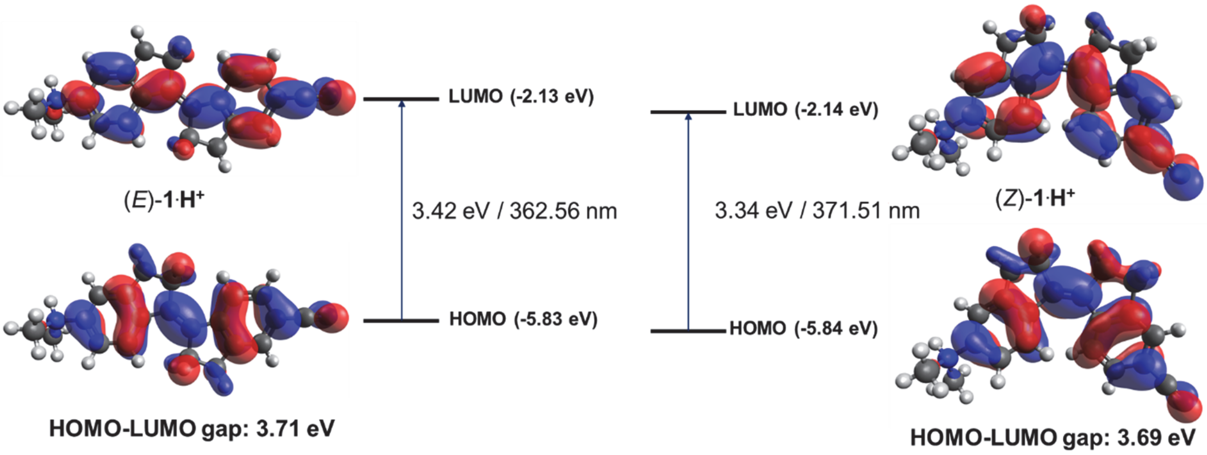

Figure S28. Description and visualization of first excited state of (*E*)-**1**·H$^+$ and (*Z*)-**1**·H$^+$ described by an excitation from HOMO (wavefunction 80) to LUMO (wavefunction 81). Molecular orbitals are visualized with an isosurface value of 0.02000. The $S_0 \rightarrow S_1$ excitation energies are listed alongside the arrows, representing the optical gaps. The HOMO-LUMO gaps are listed below.

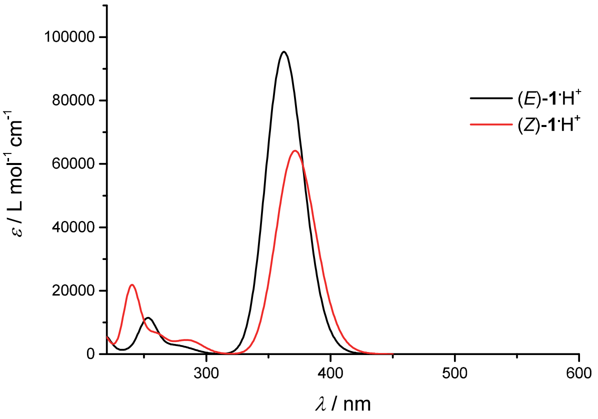

Figure S29. (A) Computed UV-Vis absorption spectra [B3LYP 6-31G(d,p) PCM (CH3CN)] plotted with peak half-width at half height set to 0.167 eV of (*E*)-**1**·H$^+$ and (*Z*)-**1**·H$^+$.

(*E*)-5	Symmetry	eV	nm	Oscillator strength	$\langle S^2 \rangle$	Wavefunctions	coefficients
1	A	3.76	329.44 nm	0.9395	0.00	62 → 63	0.70331

(*Z*)-5	Symmetry	eV	nm	Oscillator strength	$\langle S^2 \rangle$	Wavefunctions	coefficients
1	A	3.64	340.21	0.6184	0.00	62 → 63	0.70238

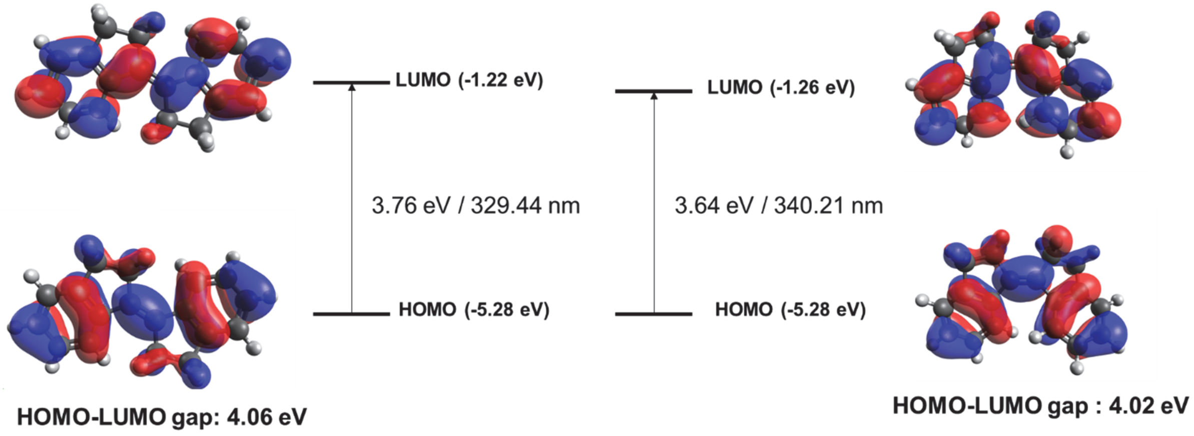

Figure S30. Description and visualization of first excited state of unsubstituted stiff-stilbene (*E*)-**6** and (*Z*)-**6** described by an excitation from HOMO (wavefunction 62) to LUMO (wavefunction 63). Molecular orbitals are visualized with an isosurface value of 0.02000. The $S_0 \rightarrow S_1$ excitation energies are listed alongside the arrows, representing the optical gaps. The HOMO-LUMO gaps are listed below.

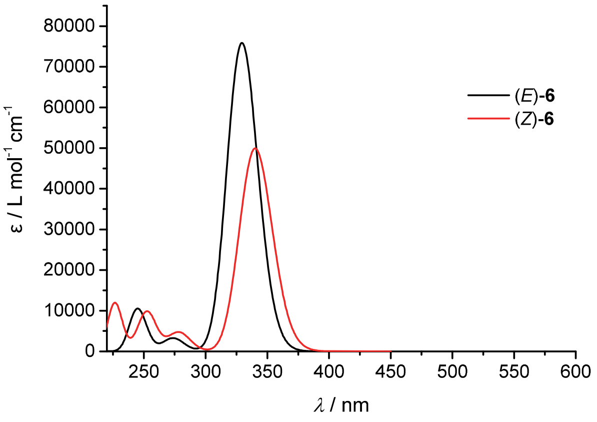

Figure S31. (A) Computed UV-Vis absorption spectra [B3LYP 6-31G(d,p) PCM (CH3CN)] plotted with peak half-width at half height set to 0.167 eV for (*E*)-**6** and (*Z*)-**6**.

References

[1] M. Oelgemöller, R. Frank, P. Lemmen, D. Lenoir, J. Lex, Y. Inoue, *Tetrahedron.* **2012**, *68*, 4048-4056.

[2] This procedure is similar to the one reported for the 6,6'-regioisomer: S. J. Wezenberg, B. L. Feringa, *Org. Lett.* **2017**, *19*, 324-327.

[3] The synthesis and characterization of the (*E*)-isomer has previously been described: M. P. O'Hagan, P. Peñalver, R. S. L. Gibson, G. C. Morales, M. C. Galan, *Chem. Eur. J.* **2020**, *26*, 6224-6233.

[4] M. Montalti, A. Credi, L. Prodi, M. T. Gandolfi, *Handbook of Photochemistry, Third Edition.*, CRC Press, Boca Raton, Fl, **2006**.

[5] C. G. Hatchard, C. A Parker, *Proc. Roy. Soc.* **1956**, *A235*, 518-536.

[6] Gaussian 09, Revision D.01, M. J. Frisch, G. W. Trucks, H. B. Schlegel, G. E. Scuseria, M. A. Robb, J. R. Cheeseman, G. Scalmani, V. Barone, B. Mennucci, G. A. Petersson, H. Nakatsuji, M. Caricato, X. Li, H. P. Hratchian, A. F. Izmaylov, J. Bloino, G. Zheng, J. L. Sonnenberg, M. Hada, M. Ehara, K. Toyota, R. Fukuda, J. Hasegawa, M. Ishida, T. Nakajima, Y. Honda, O. Kitao, H. Nakai, T. Vreven, J. A. Montgomery, Jr., J. E. Peralta, F. Ogliaro, M. Bearpark, J. J. Heyd, E. Brothers, K. N. Kudin, V. N. Staroverov, T. Keith, R. Kobayashi, J. Normand, K. Raghavachari, A. Rendell, J. C. Burant, S. S. Iyengar, J. Tomasi, M. Cossi, N. Rega, J. M. Millam, M. Klene, J. E. Knox, J. B. Cross, V. Bakken, C. Adamo, J. Jaramillo, R. Gomperts, R. E. Stratmann, O. Yazyev, A. J. Austin, R. Cammi, C. Pomelli, J. W. Ochterski, R. L. Martin, K. Morokuma, V. G. Zakrzewski, G. A. Voth, P. Salvador, J. J. Dannenberg, S. Dapprich, A. D. Daniels, O. Farkas, J. B. Foresman, J. V. Ortiz, J. Cioslowski,, D. J. Fox, Gaussian, Inc., Wallingford CT, 2013.

[7] Avogadro: an open-source molecular builder and visualization tool. Version 1.2.0. http://avogadro.cc/
